# Implication of Genetic Deletion of *Wdr13* in Mice: Mild Anxiety, Better Performance in Spatial Memory Task, with Upregulation of Multiple Synaptic Proteins

**DOI:** 10.3389/fnmol.2016.00073

**Published:** 2016-08-30

**Authors:** Shiladitya Mitra, Ghantasala S. Sameer Kumar, Vivek Tiwari, B. Jyothi Lakshmi, Suman S. Thakur, Satish Kumar

**Affiliations:** Council of Scientific and Industrial Research - Centre for Cellular and Molecular BiologyHyderabad, India

**Keywords:** *Wdr13*, mouse models, synaptic genes, memory, proteomics, behavior

## Abstract

*WDR13* expresses from the X chromosome and has a highly conserved coding sequence. There have been multiple associations of *WDR13* with memory. However, its detailed function in context of brain and behavior remains unknown. We characterized the behavioral phenotype of 2 month old male mice lacking the homolog of *WDR13* gene (*Wdr13*^−/0^). Taking cue from analysis of its expression in the brain, we chose hippocampus for molecular studies to delineate its function. *Wdr13*^−/0^ mice spent less time in the central area of the open field test (OFT) and with the novel object in novel object recognition test (NOR) as compared to the wild-type. However, these mice didn't show any significant changes in total time spent in arms or in frequency of arm entries in elevated plus maze (EPM). In the absence of *Wdr13*, there was a significant upregulation of synaptic proteins, viz., SYN1, RAB3A, CAMK2A etc. accompanied with increased spine density of hippocampal CA1 neurons and better spatial memory in mice as measured by increased time spent in the target quadrant of Morris water maze (MWM) during probe test. Parallel study from our lab has established c-JUN, ER α/β, and HDAC 1,3,7 as interacting partners of WDR13. WDR13 represses transcription from AP1 (c-JUN responsive) and Estrogen Receptor Element (ERE) promoters. We hypothesized that absence of *Wdr13* would result in de-regulated expression of a number of genes including multiple synaptic genes leading to the observed phenotype. Knocking down *Wdr13* in Neuro2a cell lines led to increased transcripts of *Camk2a* and *Nrxn2* consistent with *in-vivo* results. Summarily, our data provides functional evidence for the role of *Wdr13* in brain.

## Introduction

WDR13 belongs to a class of WD (tryptophan-aspartate) repeat-containing proteins. Human *WDR13* gene was discovered and characterized by Singh et al. (2003) and its highly conserved mouse homolog (*Wdr13*) by Suresh et al. (2005). It was independently discovered in mice by D'Agata et al. (2003) as *Cmrg1* (Cerebellar memory related gene 1). Both human and mouse homologs of this gene localize on the X chromosome and encode a protein comprised of 485 amino acids. Western blot analysis shows two major isoforms of WDR13, one near the predicted molecular weight of 53 kDa and another smaller 43 kDa band corresponding to a truncated 394 amino acid protein (Singh et al., 2015a). WDR13 is a nuclear protein without any consensus nuclear localization signal (Suresh et al., 2005). To elucidate its function, a gene-knockout mouse was generated in our laboratory. The absence of this gene in mice (*Wdr13*^−/0^) resulted in age-dependent mild obesity, pancreatic beta cell hyper-proliferation, subsequent hyper-insulinemia (Singh et al., 2012) and improvement of metabolic phenotype in *Lepr*^(db/db)^ mice (Singh et al., 2015a).

*Wdr13* gene is expressed in most tissues with relatively higher expression observed in the brain, pancreas, ovaries, and testes (Suresh et al., 2005). Research from various groups indicates the possible involvement of *Wdr13* in brain function. D'Agata et al. (2003) implicated its function in learning and memory based on the association between expression of this gene to classical conditioning of rabbit nictitating membrane response. *Wdr13* transcript has been shown to be enriched following synaptogenic lesion of the hippocampus in rats, suggesting its role as a possible neuro-protective molecule (Price et al., 2003). *WDR13* had also been reported to be associated with the phenotype of hyperactivity, learning and visual-spatial difficulties of an 11-year-old boy having approximately 1.3 Mb duplication at locus Xp11.23p11.3 (El-Hattab et al., 2011).

These reports, however, were based on correlation and did not give any direct evidence of brain specific function of *WDR13*. In the current work, we have delineated the role of this gene in brain by studying behavioral and molecular changes in *Wdr13* knockout male mice (*Wdr13*^−/0^).

## Materials and methods

### Animal experiments and handling

Mice were procured from the central Animal House facility (CCMB). They were housed in polypropylene cages with shredded corn-cob bedding with 12-h light and dark cycle (6 a.m.–6 p.m. light cycle). The required numbers of mice were generated by crossing of wild-type and mutant (heterozygous, *Wdr13*^+/−^) mice. Unless mentioned specifically, all behavior and molecular data corresponds to male *Wdr13*^−/0^ and wild-type mice in CD1 genetic background. CCMB Institutional Animal Ethics Committee approved all the animal experiments (Reg. No. CPCSEA 20/1999).

### Analysis of brain metabolism

Nuclear Magnetic Resonance (NMR) was used to analyze changes in brain metabolites of *Wdr13*^−/0^ and wild-type mice at 10 and 2 months of age. Metabolic measurements of cortex and subcortex were carried out on one group of mice containing wild type and *Wdr13* knockout mice of age 10 months (*n* = 5, 6) by co-infusion of [U-13C6]-Glucose and [2-13C]-Acetate through the tail vein. 13C labeling of amino acids in brain tissue extract was analyzed using 1H-[13C] and 13C-[1H]-NMR spectroscopy. The protocol has been described by previous studies (Patel et al., 2001; Shameem and Patel, 2012). Two month old (*n* = 4) mice cortex was analyzed for metabolic changes using infusion of [1,6-13C2]Glucose for 10 min using the methodology described earlier (Shameem and Patel, 2012; Tiwari and Patel, 2012).

### RNA *in situ* hybridization (RISH)

Dig-labeled RNA probes for anti-sense and sense strands of full length *Wdr13* cloned in pGMT vector were prepared according to instructions provided by ROCHE. RISH was carried out on cryo sections (30 μM) derived from formaldehyde perfused and fixed brain tissue from wild-type and *Wdr13*^−/0^ mice as described previously (Singh et al., 2015b). Images were taken using AxioImager2 (Zeiss) with Apotome.

### Behavioral analysis of *Wdr13*^−/0^ mice

Mice were tested for anxiety, depression, learning and memory. Video tracking software Noldus Ethovision 3.1 was used to record the movements of the mice in the tests. Litter-mates were utilized as control wild-type for different behavioral experiments. *Wdr13*^−/0^ and wild type mice in outbred CD1 background was utilized for all behavioral studies. Few of the following studies were also performed in inbred C57BL/6J background to rule out possibilities of any strain-specific phenotypes.

- Open Field Test: This test was designed to study emotionality in rats (Hall, 1934) and later reproduced in mice (Christmas and Maxwell, 1970). This test has since been performed to assess the degree of anxiety and locomoter activity in mice. The Open Field test (OFT) arena is an open square box 50 × 50 cm. The box is virtually demarcated into a central zone and peripheral zones. The experimental mouse was placed in one corner of the box and allowed to explore the arena for 5 min. The amount of time the mouse spent in the center and periphery zones within 5 min was then noted. Mice with higher anxiety levels tend to spend more time in the periphery and less time in the central area.
- Elevated Plus Maze test: This test too was performed to assess the degree of anxiety in mice (Crawley, 2006). The setup consists of a four armed maze kept on an elevated platform. The maze consists of two open arms and two closed arms (walls on the side). The mouse was kept at the center of the maze facing the open arm and allowed to explore the arena for 5 min. The amount of time spent by the mouse in the open and closed arms was then calculated. The frequency of visit to each arm was also calculated. Mice with higher anxiety levels tend to spend more time in the closed arms and less in the open arms (thigmotaxis).
- Forced Swim Test: Also known as the Porsolt Swim Test (Porsolt et al., 1977), this test is conducted to analyse depression in rodents. In this test the mice were placed in a beaker filled with water till 20–25 cm and the total duration of immobility was measured. The experiment was video-recorded and analyzed post-recording. Usually a mouse with depression phenotype remains immobile for a longer time (Can et al., 2012).
- Novel Object Recognition Test: This test had been developed to measure cognition, anxiety and preference for novelty in rodents (Antunes and Biala, 2012). In this experiment, the mice were placed in an open field on the first day. On the second day, two similar objects were introduced in the arena and the mice were habituated to them. On day three, a novel object of different shape and size replaced one of the objects. The discrimination by the mice between two objects was noted (measured as ratio of difference between time spent near novel object and familial object to that of total time spent exploring both objects). Normally, a wild type mouse prefers to explore novel objects.
- Hot Plate test: This test is generally used to analyze proper functioning of neuromuscular junction and pain sensitivity (Minett et al., 2012). The mice were placed on a hot plate at 55°C and the latency to the first jump was noted.
- Morris water maze (MWM): This experiment was developed for learning and memory analysis in rodents by Morris (1981). There are many variants of the method for training and analysis (Vorhees and Williams, 2006). Here the modified version of the Morris Water Maze (MWM) has been followed. The cues such as a tripod stand, a hanging bucket and specific arrangement of curtains were located outside a tub of 90 cm in radius and 40 cm in depth. The mouse was first placed on the submerged platform for 30 s on the first day and then taken out. In the second trial on day one, the mice were placed in water and then guided to the submerged platform. Usually each mouse were subjected to 2–3 trials in 1 day. The mice in subsequent trials were placed in water and time to reach the platform was noted. If it was unable to reach, it was guided at the end of the trial, which lasted 1 min 30 s. At the end of the trial, when the mouse reached the platform, it was left there for 15–30 s. Latency to reach the platform was noted and this was taken as an indication of learning capacity of the mice. After 5–6 days when the mice learnt to reach the platform placed anywhere in the tub, probe test was conducted 24 h post last learning trial.
  For re-learning experiment, the position of the platform was shifted to a new location after mice learnt to reach in its initial location. The learning ability of the mice to reach the platform in its new location was analyzed over multiple trials.
  In the probe test, the platform was removed and the mice were placed in the tub. The time it spends in the quadrant in which the platform was kept was noted. Repeated (extinction) trials were performed 24 h after the final learning trial. In an extinction trial the mice were subjected to probe trials from random location multiple times. Normally the probability for a wild type to search for the platform decreases over trials (Maei et al., 2009; Terry, 2009).
  For long-term memory test, probe trial was not conducted immediately after the learning trial, but mice were subjected to a probe trial after a period of 20 days using the same setup and location of cues. Time spent by the mice in the target quadrant was noted.

#### Cohorts used

Multiple cohorts were used for the afore-mentioned behavioral experiments (Table 1). Multiple experiments were executed in duplicates to validate the phenotype observed. Initially, a cohort of CD1 mice (*n* = 5) was utilized to perform OFT, Elevated Plus Maze (EPM), Novel Object Recognition test (NOR), and MWM. Between the non-stress experiments of OFT, EPM, NOR and that of MWM, there was an interval of 2 weeks. An independent cohort of CD1 mice (*n* = 17) was then subjected to OFT, EPM, and Forced Swim Test. A separate cohort of mice (*n* = 9, 10/8) was used to re-perform NOR and Hot Plate test. For MWM, we repeated learning trials and probe test in a separate cohort of mice (*n* = 6). Two other independent cohorts were utilized separately for re-learning experiment and long-term retention test in MWM (*n* = 6 each). We have also repeated FST with a separate cohort of mice (*n* = 6; data not shown).

**Table 1:** **List of Cohorts of mice utilized in behavioral experiments**.

**Strain**	**Cohort number**	***n***	**Behavioral experiments**
CD1	1	5	OFT, EPM, NOR, MWM
	2	17	OFT, EPM, FST
	3	9, 10/8	NOR, Hot Plate Test
	4	6	MWM learning and probe trials
	5	6	MWM relearning trials
	6	6	MWM long term retention
	7	6	FST (data not shown)
C57Bl/6J	1′	16	OFT
	2′	8	EPM, MWM

*OFT, Open Field Test; EPM, Elevated Plus Maze; FST, Forced Swim Test; MWM, Morris Water Maze; NOR, Novel Object Recognition*.

We used two separate cohorts for performing behavioral tests in C57Bl/6J mice. One cohort of mice (*n* = 16) was tested in OFT. Another (*n* = 8) was used to perform EPM and MWM.

### Proteomic analysis

Sample preparation, labeling, running of samples in LC-MS/MS and analysis were performed as described below. The mass spectrometry proteomics data along with the list of proteins quantified have been deposited to the ProteomeXchange Consortium via the PRIDE partner repository with the dataset identifier PXD002466.

#### iTRAQ 4 plex

We pooled hippocampi from three wild-type and three knockout (*Wdr13*^−/0^) mice for the present study. Proteins were extracted from tissue using 0.5% SDS and were estimated using Bicinchoninic acid assay (BCA) method. Two hundred micrograms of protein from both groups was taken as a starting amount. The protein from each group was treated with 2 μL of reducing agent [tris (2-carboxyethyl) phosphine (TCEP)] at 60°C for 1 h and the samples were alkylated using 1 μL of cysteine blocking reagent, methyl methanethiosulfonate (MMTS) for 10 min at RT. After alkylation, the samples were digested with trypsin (Sequencing Grade Modified Trypsin, Promega Cat#:V511A) using 1:20 (w/w) at 37°C for 16 hrs. We split the samples based on the protein amount (100 μg) in each group and labeled with iTRAQ 4plex (catalog # 4352135, Applied Biosystems, Foster City, CA, USA) reagents as per manufacturer's protocol. In iTRAQ 4-plex experiments, peptides from wild type were labeled with 114 and 115 tags, while knockout samples were labeled with 116 and 117 tags. After labeling, we pooled the samples and carried out desalting using C18 spin columns (89873—Pierce® C18 Spin Columns) as per manufacturer's protocol. Later, the iTRAQ 4-plex labeled samples were processed further for LC-MS/MS analysis.

#### LC-MS/MS analysis

Samples were analyzed on UPLC (Dionex The UltiMate® 3000 HPLC) interfaced with Q-Exactive mass spectrometer (Thermo Scientific, Bremen, Germany). Trypsin digested peptides were loaded on a 15 cm long column (EASY-Spray column ES800, 15 cm × 75 μm ID, PepMap C18, 3 μm). Column was heated at 30°C with integrated temperature control. Peptides were separated using linear gradient from 2 to 98% of buffer B (95% acetonitrile and 0.1% formic acid) at a flow rate of 300 nl/min, which was followed by a column re-equilibration reaching 2% of buffer B for few minutes. Gradient length had been adjusted to 50 min. The acquisition of the data was carried out using Xcalibur 2.1 (Thermo Scientific, Bremen, Germany). MS spectra were acquired in a data dependent manner in the range of m/z 350–1800 at a scan resolution of 70,000 and followed by top 10 precursor ions selected for MS/MS analysis at a scan resolution of 17,000. Normalized collision energy (NCE) was set to 27 for fragmentation. The priority of the precursor ion selection was based on the charge state in the order of 2+, 3+ and >3+. Unassigned and single charge state precursor ions were excluded from fragmentation. The dynamic exclusion was set as 30 s during data acquisition. The nano source was operated with 2.2 KV and the capillary temperature at 300°C. Isolation width has been adjusted.

#### Data analysis

The acquired data was analyzed using Proteome Discoverer 1.3 (Thermo Scientific, Bremen, Germany) software. We used International Protein Index (IPI) (version 3.83, Mouse) database to search for peptides. The workflow created included spectrum files, spectrum selector and Sequest. Search nodes were given as searches including peptide validator for false discovery analysis and used a reporter ion quantifier for quantitation. We set the parameters, which included Methylthio (C), iTRAQ label at N-terminus of the peptide and lysine as fixed modifications. Oxidation of methionine (M) and deamidation(N/Q) were used as variable modification. The parent and fragment mass error tolerance were set as 20 ppm and 0.2 Da respectively. We acquired a total of 13,059 MS/MS scans. We calculated false discovery rate (FDR) by enabling the peptide sequence analysis using decoy database and top ranked hit based on peptide score, XCorr for Sequest. We applied 1% FDR in our analysis and proteins with a minimum of 1 unique peptide were considered.

### Golgi cox staining

Golgi cox staining was done on 100 μM brain sections using a protocol described previously (Chakravarty et al., 2015). Six mice, each of wild-type and *Wdr13*^−/0^ were selected for the analysis. A minimum of six CA1 neurons from hippocampal sections for each mouse was analyzed for spine density. Spines from the apical region of CA1 neurons were considered for the analysis. Spine density was analyzed in dendritic sections of 10 μm length. In total, about 90–100 spines were analyzed for each genotype. A semi-automated procedure for calculating spine density was utilized as described previously using FIJI and Image J (Orlowski and Bjarkam, 2012). In brief, high-resolution images of spines were converted to 8 bit image using ImageJ and threshold applied. Areas to be analyzed were selected, pasted in a new document and were converted to binary. These were then skeletonized using ImageJ or FIJI and analysis of the skeleton was performed. For analyzing dendritic branching, 6–8 CA1 neurons from five each of wild-type and *Wdr13*^−/0^ mice were traced using NeuronJ plugin and Sholl analysis was performed using Sholl Analysis Plugin (v 1.0) for ImageJ. Images were taken using AxioImager2(Zeiss) with Apotome.

### BrDU labeling and counting

Mice were injected with 200 mg/kg (body weight) of BrDU, 24 h prior to processing and analysis. BrDU injections were given to mice either in resident conditions or at the final day of learning trials of MWM. Brains were collected while whole body perfusion. Serial coronal sections of 30 μm each were obtained from a single brain encompassing SVZ (Sub-ventricular Zone) and SGZ (Sub Granular Zone) or DG (Dentate Gyrus) and every 6th section (excluding the early and late formations) were processed for immunohistochemistry using a protocol described previously (Becker et al., 2008). Similar sections from *Wdr13*^−/0^ and wild-type were stained for BrDU incorporation[Anti-BrDU (Sigma, B8434) 1:300] by fluorescent labeling or DAB (Invitrogen) staining according to a previously described protocol (Singh et al., 2012, 2015b) or manufacturer's instructions and counting of one hemisphere was carried out. Area of each DG per section was analyzed using ImageJ and BrDU counts were normalized for the median areas of the sections (DG). A minimum of 5 mice and 6 s from each mouse has been used for the experiment.

### Cell culture and transfections

Neuro2a cells were cultured in DMEM media with 10% Fetal Bovine Serum supplemented with antibiotics. Neuro2a cells grown in 24 well plates were assayed for effect of WDR13 on the expression of luciferase from AP1 and ERE site containing promoters using protocol previously described (Singh et al., 2015b). For analysis of luciferase from AP1 site containing promoter, Flag-WDR13 was co-transfected with c-JUN at (150:150) ng and (200:150) ng along with AP1-Luciferase and in Neuro2a cells in 24 well plate. For analysis of luciferase from ERE site containing promoter, Flag-WDR13 was transfected at 150 ng along with ERE-luciferase and in Neuro2a cells grown in 24 well plate, with or without supplementation of 10 nM Estradiol. Neuro2a cells used for assaying luciferase activity from ERE-promoter were maintained in DMEM media with 10% charcoal stripped serum. Transfection was carried out using Lipofectamine LTX (Life Technologies) as per manufacturer instructions. The results obtained from luciferase activity were normalized to β-galactosidase activity (indicative of transfection efficiency). *Wdr13* siRNA (Sc-155258-Santacruz) and control scrambled RNA (Sc-37007) (Santacruz) were introduced in Neuro2a cell line using RNAiMax (Invitrogen) at 100 nM as per manufacturer's instructions and previous reports (Singh et al., 2012). All the experiments were conducted in triplicates.

### RNA isolation, primer designing, and real time analysis

The primers were designed using the Primer3 software and PrimerBank. RNA was isolated using Trizol™ method and cDNAs were prepared using the protocol described by Sambrook and Russell (2001), and other previous studies (Singh et al., 2012, 2015b). Briefly, hippocampi were homogenized in Trizol™ and 1/10th volume of 1-Bromo 3- Chloropropane (Sigma) added to the solution. Aqueous phase was collected after centrifugation and RNA precipitated using equal volumes of isopropanol. After ethanol wash, RNA was air-dried and dissolved in DEPC treated water. Real time PCR was performed using SYBR green 2X mix (Invitrogen and Thermo-Fischer). ABI Prism SDS 7000 and ABI 3900 HT were used to perform real time PCR as per company protocol.

Primer sequences:

**Table:** 

**Gene name**	**Forward (5′–3′)**	**Reverse (3′–5′)**
*Camk2a*	TGCCTGGTGTTGCTAACCC	CCATTAACTGAACGCTGGAACT
*Gria1*	TCCCCAACAATATCCAGATAGGG	AAGCCGCATGTTCCTGTGATT
*Gria2*	GCCGAGGCGAAACGAATGA	CACTCTCGATGCCATATACGTTG
Grin1	AGAGCCCGACCCTAAAAAGAA	CCCTCCTCCCTCTCAATAGC
*Grin2a*	ACGTGACAGAACGCGAACTT	TCAGTGCGGTTCATCAATAACG
*Nrgn*	CAAACCCCATACTCCCAAAA	ACGAAAGGACTTGGTGGTTG
*Nrxn2*	GCTCTGCATCCTCATTCTCC	TGTTCTTCTTGGCCTTGCTT
*Rab3a*	GTGGGCAAAACCTCGTTCCT	TCCTCTTGTCGTTGCGGTAGA
*Syn1*	AGCTCAACAAATCCCAGTCTCT	CGGATGGTCTCAGCTTTCAC

### Western analysis

Protein was isolated from hippocampi after homogenizing the tissues in SDS lysis buffer (Sambrook and Russell, 2001). Western Blotting was performed using manufacturers protocol or protocol previously described (Singh et al., 2012, 2015b) with antibody against WDR13 [(HPA000913, Sigma) 1:1000 in 5%BSA], c-JUN [(sc-45, Santacruz) 1:500 in 5%BSA] and β-ACTIN [(sc-47778, Santacruz) 1:1000 in 5%BSA].

### Histological analysis

We performed Nissl staining and Hematoxylin and Eosin (H&E) staining on brain cryo-sections (30 μm) of 4 month old wild-type and *Wdr13*^−/0^ mice to understand any gross changes in anatomy. For H&E staining (Fischer et al., 2008), the sections were air-dried and then immersed in Hematoxylin solution followed by Eosin solution with washes in water. The sections were then dehydrated using a gradient of alcohol concentrations, washed in xylene and mounted. For Nissl staining (Nissl, 1894), air-dried cryo-sections were de-fatted using xylene and absolute alcohol before rehydrating them and immersing in 0.1% Cresyl Violet solution (containing glacial acetic acid). The sections were then washed in water, dehydrated and mounted on slides.

### Statistical analysis

One or Two way analysis of variance (ANOVA) and student's unpaired *T*-test were conducted with significance at *p* < 0.05(denoted as ^*^) and at *p* < 0.005(denoted as ^**^). For samples with *n* > 5, data are presented as mean ± SEM.

## Results

**Figure 1:**
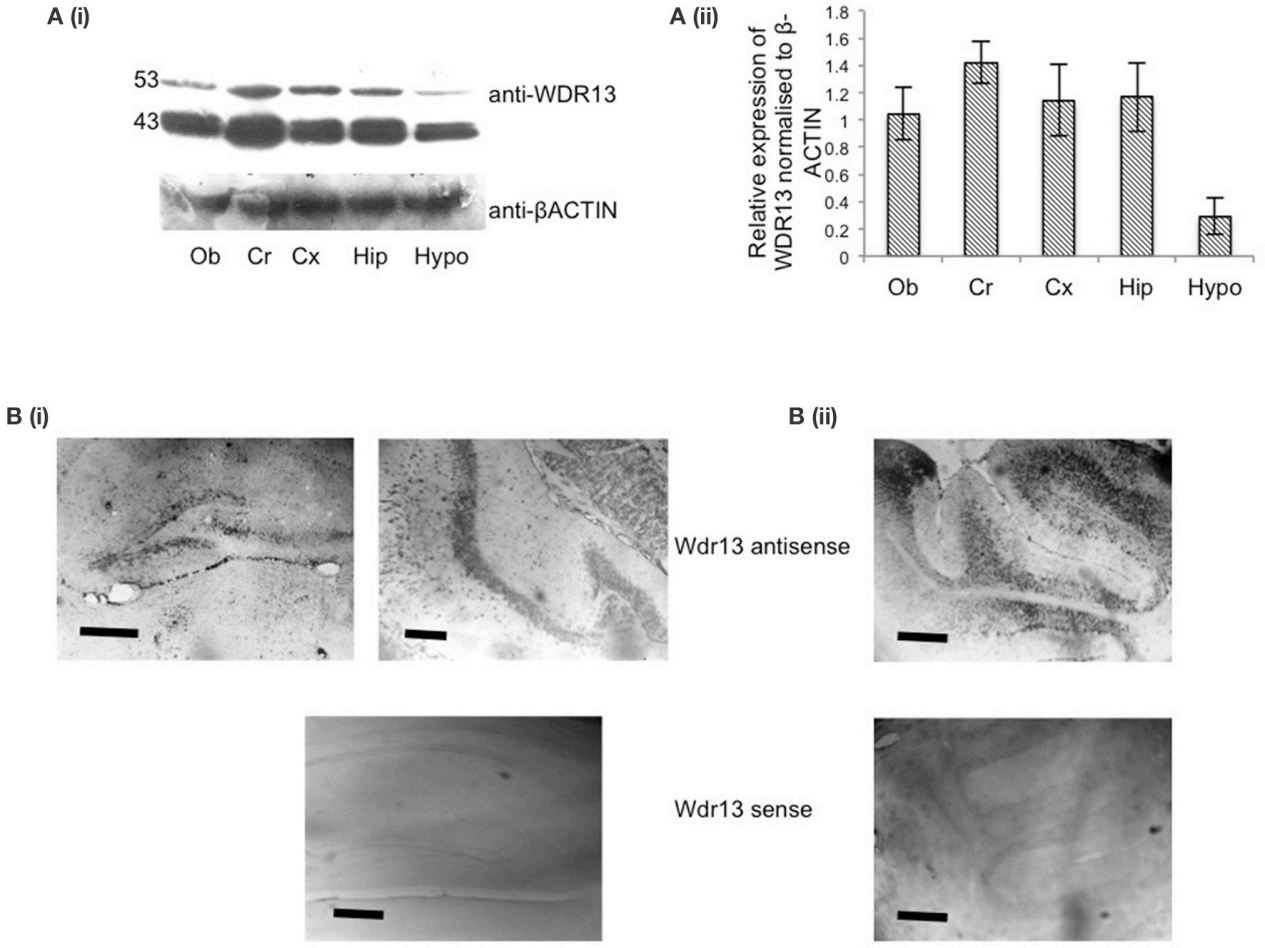
**(Ai)** Representative blot for western analysis using antibody against WDR13 in different regions of the mice brain at 2 months age (Ob, Olfactory bulb; Cr, Cerebellum; Cx, Cortex; hip, Hippocampus; Hypo, Hypothalamus). **(Aii)** Relative quantification of expression of WDR13 in different brain regions (*n* = 3). Data represented as ±SD **(B)**. RNA-*in situ* hybridization using full length anti-*Wdr13* probe showing localization in **(i)** Hippocampus and **(ii)** Cerebellum. Top panel(s) depicts sections probed using *Wdr13* antisense probe and bottom panel(s) depict sections probed using *Wdr13* sense probe. Scale: 100 μm.

Our objective was to delineate the distribution of WDR13 in the brain. Both the isoforms of WDR13—53 and 43 kDa, showed differential expression in 2 months old mice with Cerebellum (Cr) showing comparatively higher levels of expression, followed by other regions of the brain, namely, Hipocampus (Hip), Cortex (Cx), and Olfactory bud (Ob) (Figure 1A). Hypothalamus (Hypo) showed lower expression levels as compared to Cerebellum. Previous reports (D'Agata et al., 2003) and, those reported in Allen Institute of Science Mouse Brain Atlas (Lein et al., 2007) showed that *Wdr13* transcript prominently localized to Cortex, Hippocampus, Cerebellum, and Olfactory Bulb. We performed RNA *in-situ* hybridization (RISH) using antisense probe against full length *Wdr13* and found similar results (Figure 1B, Supplementary Figure 1). Considering its expression and previous reports of its association with memory (D'Agata et al., 2003), we selected hippocampus for our subsequent analysis.

We also compared whole brains from wild-type and *Wdr13*^−/0^ mice to investigate any notable differences. Analysis of the brains did not reveal any significant differences in weight (Singh et al., 2012) or shape. We also did not find any significant differences (*n* = 3) in the gross anatomy of the brains (in both coronal and sagittal sections) from wild-type and *Wdr13*^−/0^ mice (Supplementary Figure 2).

### Two months old *Wdr13*^−/0^ mice did not show changes in brain metabolism

*Wdr1*3^−/0^ mice exhibited changes in general metabolism with progression of age (Singh et al., 2012), which became prominent at an age of 9 months. We performed NMR analysis of brain metabolites in 2 months (*n* = 4) and 10 months age (*n* = 5, 6) to find out if there were any changes in brain metabolism, and if these were correlated to changes in systemic metabolism in *Wdr13*^−/0^ mice.

**Figure 2:**
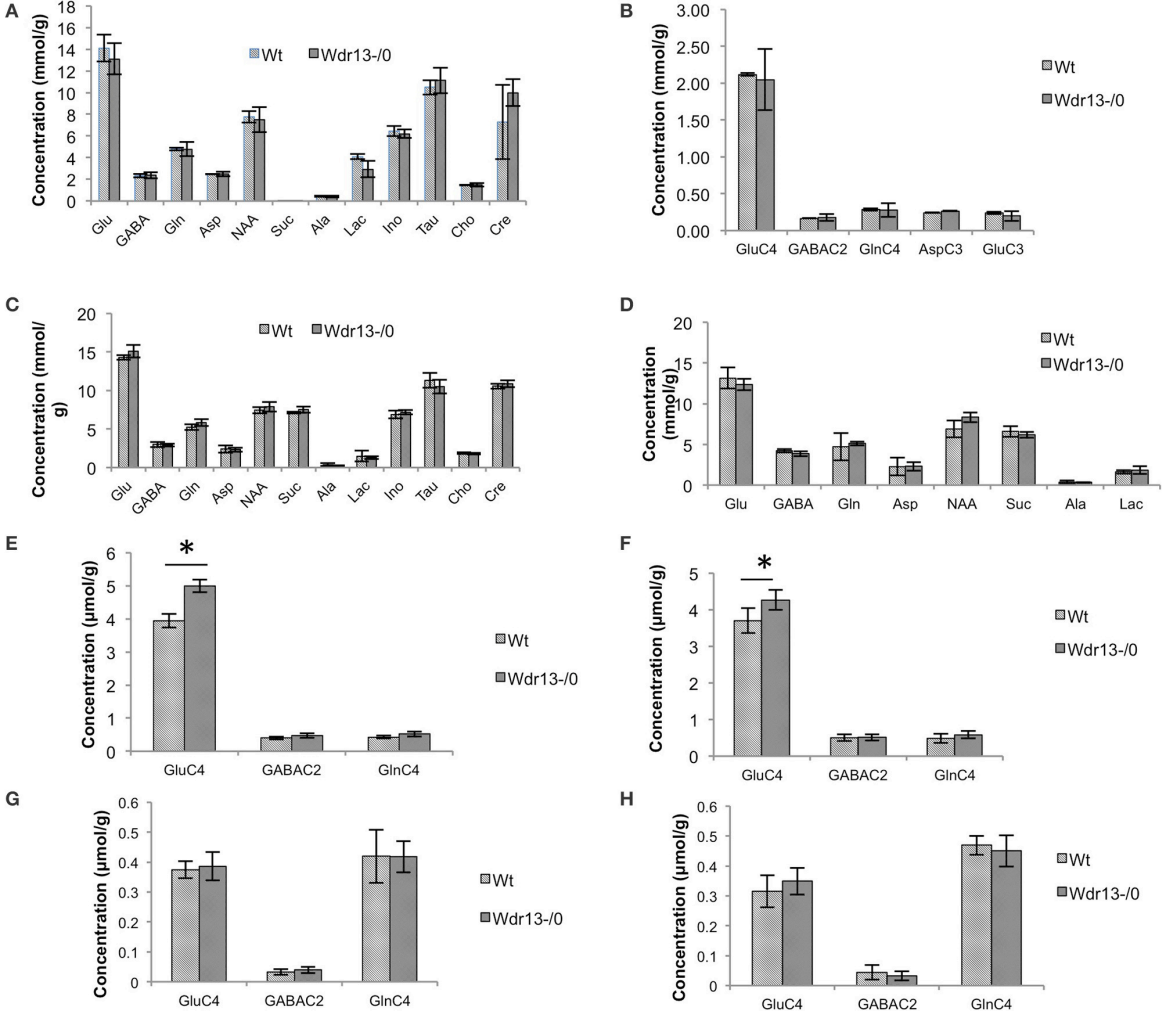
**(A,B)** Analysis of brain metabolites using NMR of 2 month old *Wdr13*^−/0^ and wild-type mice (*n* = 4) **(A)**. Concentration of metabolites in cerebral cortical extract of mice using [2-13C]glycine as reference. **(B)** 13C labeled metabolites in cortex from [1,6-(13)C(2)]glucose of 2 months old *Wdr13*^−/0^ mice showed no significant difference (*p* > 0.05) than the wild-type mice. Data is represented ±SD. **(C–H)** Analysis of brain metabolites using NMR of 10 month old *Wdr13*^−/0^ and wild-type mice (*n* = 5, 6) **(C)**. Concentration of metabolites in cerebral cortical extract of mice using [2-13C]glycine as reference. **(D)** Concentration of metabolites in cerebral sub-cortical extract of mice using [2-13C]glycine as reference. **(E)** 13C labeled metabolites enriched from [U-13C6]glucose in cortex showed significant increase (*p* < 0.05) in levels of glutamate C4 (GluC4) in *Wdr13*^−/0^ mice than the wild-type mice. **(F)** 13C labeled metabolites enriched from [U-13C6]glucose in subcortex also showed significant increase (*p* < 0.05) in levels of glutamate C4 in the *Wdr13*^−/0^ mice. **(G)** 13C labeled metabolites from [2-13C]acetate in cortex and in **(H)** Subcortex did not show any significant differences (*p* > 0.05) between *Wdr13*^−/0^ and wild-type mice. Data represented as ±SEM. Wt, wild-type; *Wdr13*^−/0^, *Wdr13* knockout mice; Glu, Glutamate, GABA; Gln, Glutamine; Asp, Aspartate; NAA, N-AcetylAspartate; Suc, Succinate; Ala, Alanine; Lac, Lactate; Ino, Inositol; Tau, Taurine; Cho, Choline; Cre, Creatinine or Cre. ^*^p < 0.05.

We found no significant changes in cortical brain metabolism in 2 months old *Wdr13*^−/0^ mice (Figures 2A,B). However, there was a significant increase in 13C concentration of glutamate (Glu) from [U-13C6]Glucose (*p* < 0.05) but not in the levels of metabolites of cortex and subcortex of *Wdr13*^−/0^ mice at 10 months of age (Figures 2C–F). There were also no significant changes (*p* > 0.05) in the 13C glutamate levels enriched from [2-13C]-Acetate (Figures 2G,H) at this age.

Glucose is utilized by both neurons and astroglia in the brain for their metabolism, whereas acetate is specifically taken up and assimilated by astrocytes (Waniewski and Martin, 1998). Labeling of Glutamate, GABA and Glutamine from [U-13C6]Glucose indicates initial estimate of the glutamatergic, GABAergic TCA cycle and total neurotransmitter cycle fluxes, respectively. Labeling of Glutamate, GABA and Glutamine from [2-13C]Acetate is representative of astroglial metabolism (Shameem and Patel, 2012). Thus, the increase in glutamate metabolism enriched from glucose but not acetate indicated it to be specific to neurons. Hence, the observed results might be primarily because of neuron specific activity of *Wdr13*. However, since brain metabolism is altered by changes in levels of circulating insulin (Bingham et al., 2002) and in obesity (Yau et al., 2012), studying *Wdr13*^−/0^ mice at 10 months might not distinguish properly between the brain-specific function of *Wdr13* and changes in systemic metabolism. Therefore, to understand the primary brain and behavior phenotype of *Wdr13*^−/0^ mice, all behavioral, molecular and histological studies were carried out at 2–3 months of age.

Based on existing literature and the pattern of expression of *Wdr13* in the brain, we analyzed *Wdr13*^−/0^ mice for any altered anxiety or cognitive function (s).

### *Wdr13*^−/0^ mice showed mild anxiety

**Figure 3:**
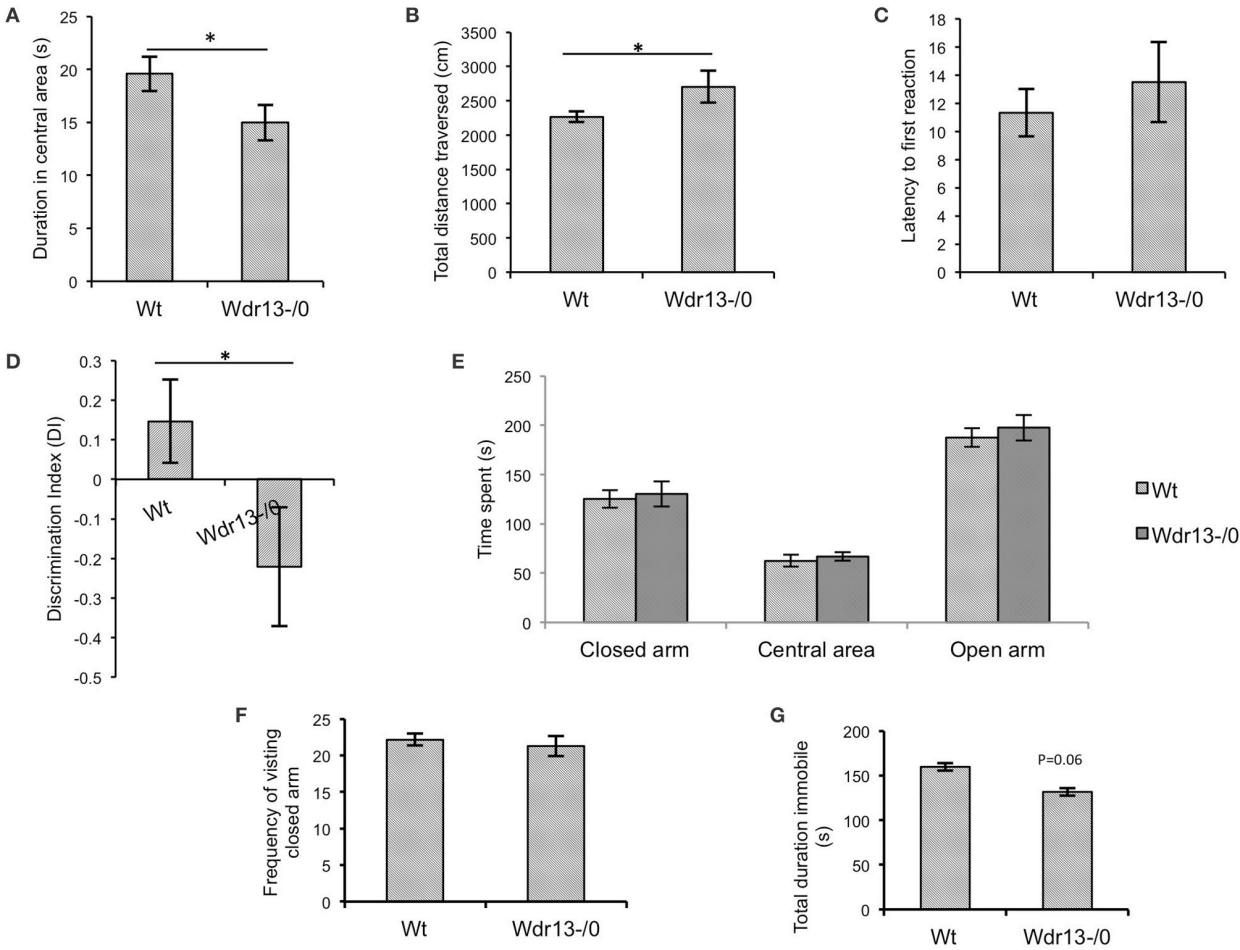
**Behavioral analysis of ***Wdr13***^**−/0**^ and wild-type mice at 2 months of age. (A)** Time spent in the central area of open field test. *Wdr13*^−/0^ mice spent significantly (*T*-test; *p* < 0.05) less time in the central area of the open field than wild-type mice (*n* = 17; Cohort 2). **(B)** Total distance traversed in open field test. *Wdr13*^−/0^ mice traveled significantly (*T*-test; *p* < 0.05) more distance in the open field arena than wild-type mice (*n* = 17; Cohort 2). **(C)** Hot plate test. There was no significant difference between *Wdr13*^−/0^ and wild-type mice (*T*-test; *p* > 0.05) in latency to react to pain sensing when placed on a hot plate (55°C) (*n* = 8; Cohort 3). **(D)** Novel object recognition test. There was a significant difference (*T*-test; *p* < 0.05) between *Wdr13*^−/0^ mice and the wild-type mice in discrimination index (*n* = 10, 9; Cohort 3). The knockout mice spent less duration exploring the novel object as compared to the wild type. **(E,F)** Elevated plus maze test. *Wdr13*^−/0^ mice did not show any significant differences (*T*-test; *p* > 0.05) than the wild-type mice in total time spent and frequency of visit to closed arms of the elevated plus maze (*n* = 17; Cohort 2) **(G)**. Forced swim test. *Wdr13*^−/0^ mice showed slightly less immobility as compared to the wild-type mice (*n* = 17; Cohort 2). This difference was however not statistically significant (*T*-test; *p* = 0.06). Data represented as ±SEM. Wt, wild-type; *Wdr13*^−/0^, *Wdr13* knockout mice. ^*^p < 0.05.

*Wdr13*^−/0^ mice in both CD1 (Figures 3A,B; *p* < 0.05; *n* = 17; Cohort 2 and Supplementary Figure 3A; *p* = 0.07; *n* = 5; Cohort 1) and C57Bl/6J (**Figures 5Ai,ii**; *p* < 0.05; *n* = 16; Cohort 1′) genetic background spent less time in the central area and traversed more distance in the OFT indicating anxiety and hyper-activity. There were no significant differences in pain response (Figure 3C; *p* = 0.26) in hot plate test (*n* = 8; Cohort 3), indicating that neuro-muscular junctions and motor response were not affected. In the NOR, *Wdr13*^−/0^ mice spent less time (Figure 3D, *p* < 0.05 *n* = 10, 9; Cohort 3 and Supplementary Figure 3B; *p* = 0.06; *n* = 5; Cohort 1) in Discrimination Index (DI) exploring the novel object and more time with the familiar object. In other words, the *Wdr13*^−/0^ mice actively discriminated against the novel object. Hence this behavioral phenotype may be associated with novelty driven anxiety, rather than the lack of ability to discriminate as the DI ≠ 0. The mutant mice however, didn't show any significant changes in total time spent and frequency of visit to arms in EPM (Figures 3E,F; *n* = 17; Cohort 2 and Supplementary Figure 3C; *n* = 5; Cohort 1; *p* > 0.05), although in C57Bl/6J background (*n* = 8; Cohort 2′), they showed a slight (*p* = 0.06) increase in frequency of visit to the closed arm [**Figures 5Bi,ii**]. In forced swim test (FST), the *Wdr13*^−/0^ mice (*n* = 17; Cohort 2 and Cohort 7) showed slightly lesser immobility (Figure 3G; *p* = 0.06) as compared to wild-type, though this was not statistically significant. All these data collectively indicated that the *Wdr13*^−/0^ mice exhibited mild anxiety.

### *Wdr13*^−/0^ mice showed better performance in hippocampal dependent spatial memory task associated with upregulated synaptic proteins

**Figure 4:**
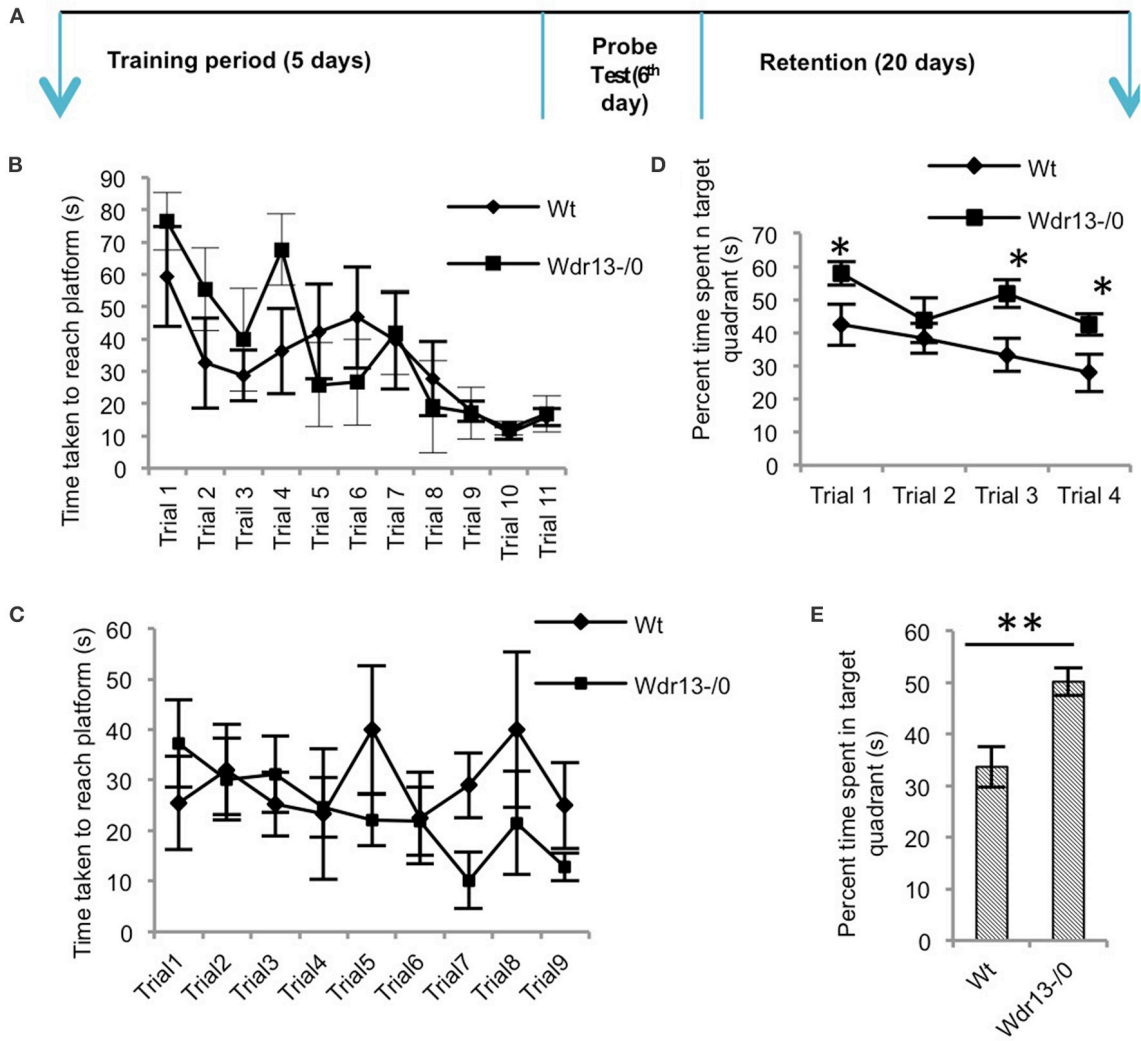
**The Morris water learning and memory test. (A)** Schematic of the protocol followed for the experiments. **(B)** There was no significant difference (ANOVA; *p* > 0.05) between *Wdr13*^−/0^ and the wild-type mice in the latency (time) to reach the platform through the learning trials (*n* = 6; Cohort 4). **(C)** Re-learning experiment showed no significant difference (ANOVA; *p* > 0.05) between the *Wdr13*^−/0^ and wild-type mice (*n* = 6; Cohort 5). **(D)** On successive (extinction) probe trials, *Wdr13*^−/0^ mice spent significantly [ANOVA, *F*_(1, 40)_ = 14.24; *p* < 0.005] more time in the target quadrant as compared to the wild-type mice (*n* = 6; Cohort 4). **(E)** *Wdr13*^−/0^ mice showed better long-term memory by spending significantly (*T*-test; *p* < 0.005) more time in the target quadrant when subjected to probe trial after 20 days of learning phase (*n* = 6; Cohort 6). Data represented as ±SEM. Wt, wild-type; *Wdr13*^−/0^, *Wdr13* knockout mice. ^*^p < 0.05, ^**^p < 0.005.

Hippocampus has been shown to be involved in spatial learning and spatial memory (Vorhees and Williams, 2006; Clark et al., 2007; Inostroza et al., 2011; Barnhart et al., 2015) and also long term retention of memory (Ramos, 2000) as assessed by MWM. Hence we employed MWM as a test to assess hippocampal dependent learning and memory behavior (Figure 4A).

**Figure 5:**
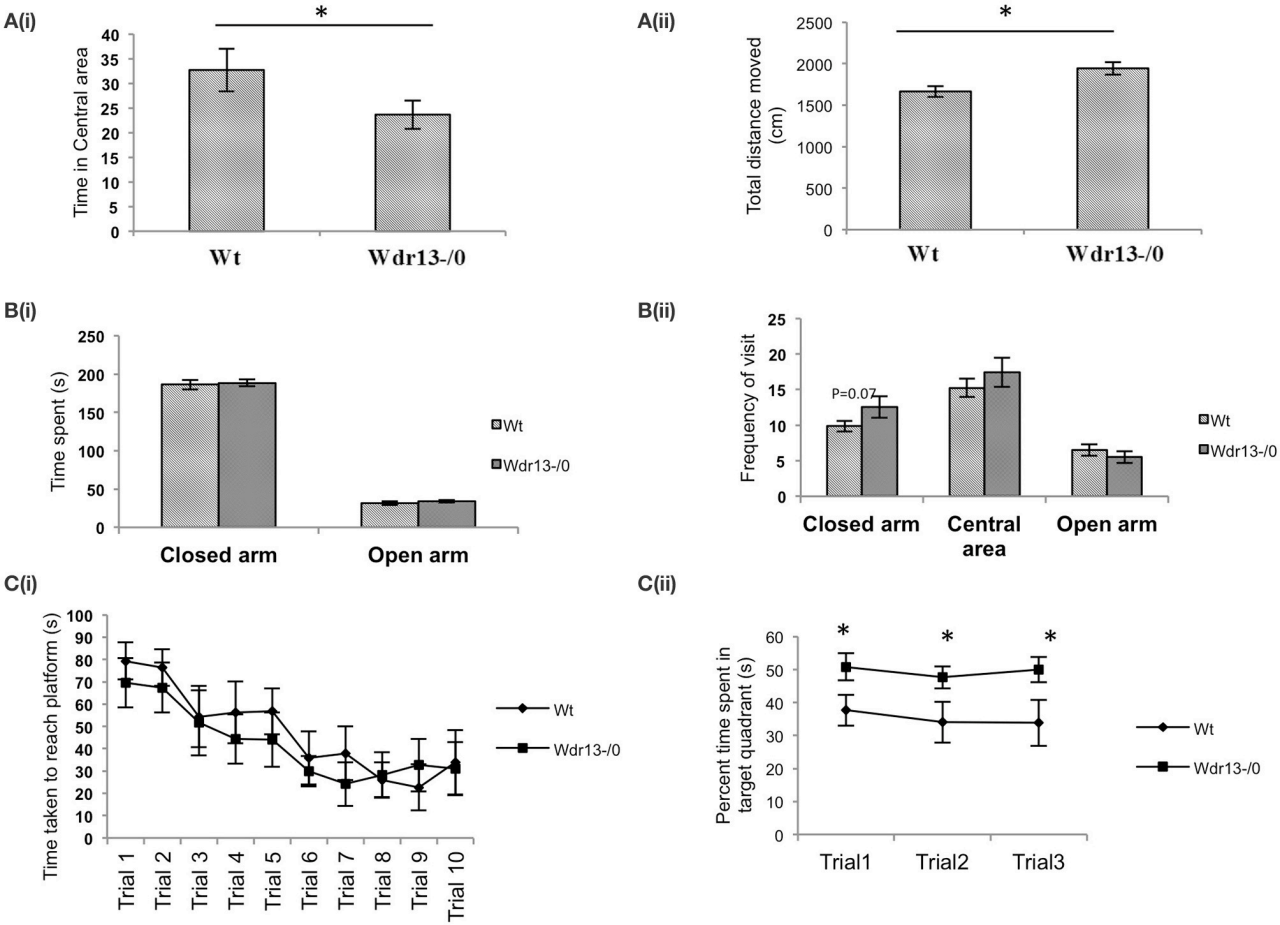
**Behavioral tests of ***Wdr13***^**−/0**^ and wild-type mice in C57Bl/6J background at 2 months of age. (A)** Open Field Test. **(i)** *Wdr13*^−/0^ mice spent significantly (*T*-test; *p* < 0.05) less time in the central area of the open field as compared to wild-type mice. **(ii)** *Wdr13*^−/0^ mice moved significantly (*T*-test; *p* < 0.05) more distance in the open field arena than the wild-type mice (*n* = 16; Cohort 1′). **(B)** Elevated plus maze. **(i)** There was no significant difference (*T*-test; *p* > 0.05) between *Wdr13*^−/0^ and wild-type mice in time spent in closed arm and central area **(ii)**. *Wdr13*^−/0^ mice showed marginally increased frequency to visit the closed arm than the wild-type mice. However, this difference was not significant statistically (*T*-test; *p* = 0.06). No significant differences (*T*-test; *p* > 0.05) were observed between the wild-type and *Wdr13*^−/0^ mice in the frequency of visit to the central area or open arm (*n* = 8; Cohort 2′). **(C)** Morris Water Learning and Memory test. **(i)** There was no significant difference (ANOVA, *p* > 0.05) between *Wdr13*^−/0^ and wild-type mice in the latency to reach the platform during the learning trials **(ii)**. *Wdr13*^−/0^ mice spent significantly [ANOVA, *F*_(1, 36)_ = 10.24; *p* < 0.005] more time in the target quadrant on repeated probe trials (extinction trials) as compared to the wild-type mice (*n* = 8; Cohort 2′). Data represented as ±SEM. Wt, wild-type; *Wdr13*^−/0^, *Wdr13* knockout mice. ^*^p < 0.05.

*Wdr13*^−/0^ mice showed no significant difference (*p* > 0.05) in the time taken to reach the platform through the learning trials [Figure 4B (CD1; *n* = 6; Cohort 4)]. Using another cohort of mice we performed re-learning experiment, when the platform was re-located after the mice were trained to reach the platform in a specific quadrant. In this experiment also *Wdr13*^−/0^ mice (*n* = 6; Cohort 5) didn't differ significantly (*p* > 0.05) from the wild-type in the time taken to reach the platform in the new location (Figure 4C). However, *Wdr13*^−/0^ mice spent more time in the target quadrant during the probe (extinction) trials {[Figure 4D; ANOVA, *F*_(1, 40)_ = 14.24; *p* < 0.005] (CD1; *n* = 6; Cohort 4)}, which were performed on the same cohort of mice which underwent learning trials. Probe trial after learning trials was also conducted on a separate cohort of mice and similar results [Supplementary Figure 3D; ANOVA, *F*_(1.56)_ = 5.44; *n* = 5; Cohort 1; *p* < 0.05] were obtained, validating the observed phenotype. Interestingly, even in a long term retention test performed 20 days after learning trials on an independent cohort of mice, the mutant (*n* = 6; Cohort 6) spent significantly more time (Figure 4E; *p* = 0.003) in the target quadrant. We found similar results using *Wdr13*^−/0^ and wild-type mice in C57Bl/6J inbred background; mutant mice did not show any significant differences in the latency to reach the platform during the learning trials [Figure 5Ci (C57Bl/6J; *n* = 8; Cohort 2′)] but spent more time in the target quadrant {[Figure 5Cii; ANOVA, *F*_(1, 36)_ = 10.24; *p* < 0.005] (C57Bl/6J; *n* = 8; Cohort 2′)} during the extinction probe trials. Thus, MWM test indicated that the *Wdr13*^−/0^ mice didn't show any differences in learning ability, but had improved retention of spatial memory in MWM than the wild-type and this phenotype was strain independent.

Singh et al. (2012) had previously shown that in *Wdr13*^−/0^ mice, there was an increase in pancreatic beta cell proliferation. We asked whether in the brain of the mutant mice, the proliferation of adult neuronal precursor cells were affected. Since, adult neurogenesis has been implicated in learning and memory (Deng et al., 2010), we also asked if the enhanced spatial memory phenotype of *Wdr13*^−/0^ mice was associated with increased neuronal proliferation induced adult neurogenesis. However, BrDU labeling of proliferating neurons in the Sub Ventricular Zone (SVZ) and Dentate Gyrus (DG) of Sub Granular Zone (SGZ) of *Wdr13*^−/0^ and wild-type mice under resident conditions showed no significant changes (Supplementary Figures 4A,A′,B,B′; *p* > 0.05). Next, we wanted to know whether absence of *Wdr13* had any effect on eural proliferation after the mice was subjected to learning tasks. Since learning tasks are shown to increase neurogenesis (Gould et al., 1999), we checked BrDU incorporation in proliferating neurons after MWM learning trials but again failed to note significant changes (Supplementary Figures 4C,D; *p* > 0.05). It may be noted that we also did not find significant difference of labeled BrDU cells between wild-type controls and those subjected to learning task. This is not a contradiction because Gould et al. (1999) also stated that the increased neurogenesis that they observed was mainly due to stability of neural progenitors labeled with BrDU before the start of the learning trials. The authors also failed to find any changes in number of cells labeled during or after the trials, which was similar to our findings. This indicated that adult neurogenesis might not have any significant role behind the observed phenotype.

**Figure 6:**
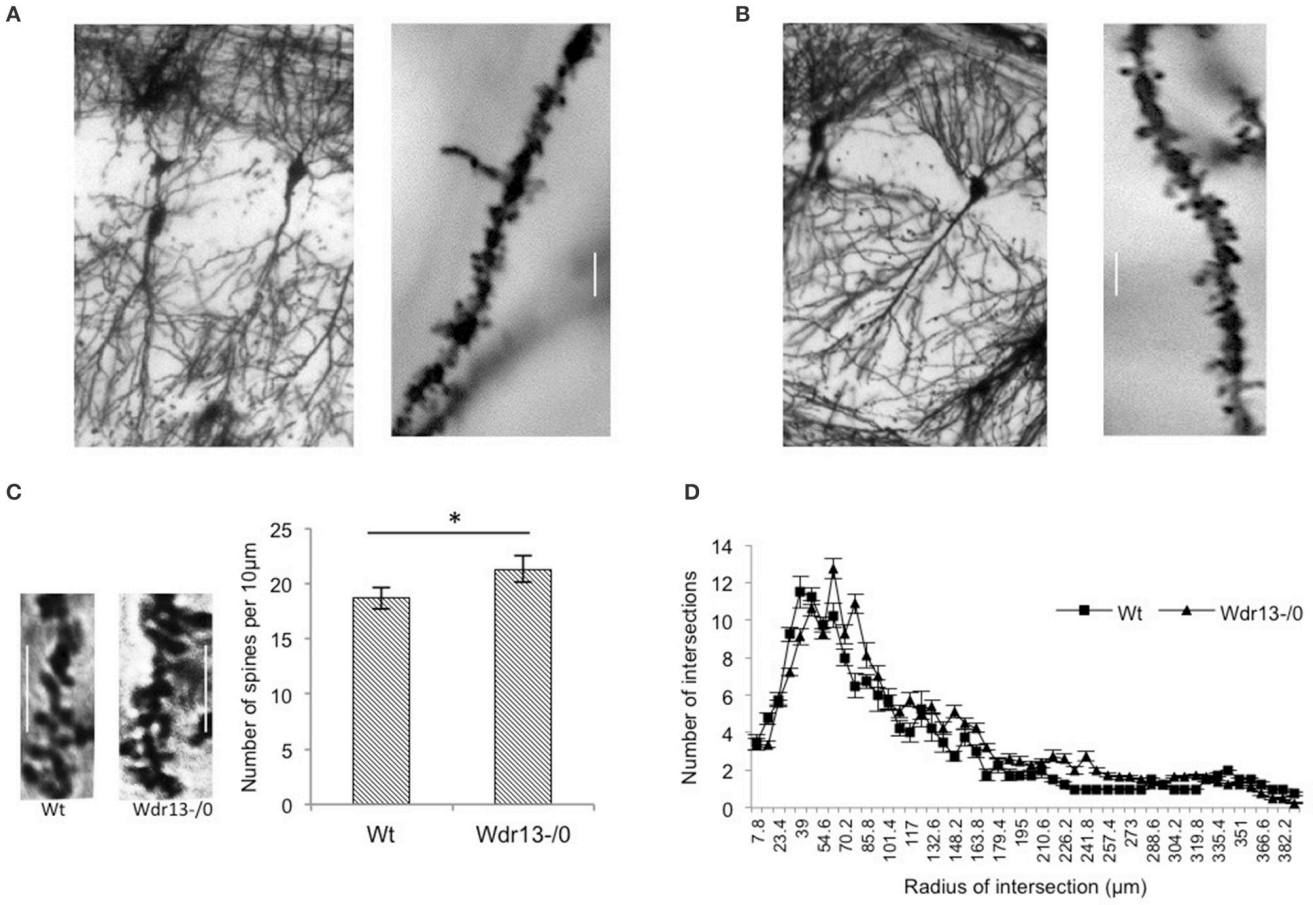
**Analysis of spine density and dendritic branching of CA1 neurons of the hippocampus from wild-type and ***Wdr13***^**−/0**^ mice. (A,B)** Representative pictures of wild-type **(left)** and *Wdr13*^−/0^ **(right)** CA1 neurons and spines. **(C)** The spine density of apical CA1 neurons was significantly (*T*-test; *p* < 0.05) higher in *Wdr13*^−/0^ mice as compared to the wild-type. Scale: 5 μm. **(D)** There was no significant difference (ANOVA; *p* > 0.05) in dendritic branching of CA1 neurons of *Wdr13*^−/0^ and wild-type mice. Data represented as ±SEM. Wt, wild-type; *Wdr13*^−/0^, *Wdr13* knockout mice. ^*^p < 0.05.

Dendritic spines have been associated with synaptic plasticity (Segal, 2005) and spine density and structures have been implicated with long and short term memory (Moser et al., 1994; Leuner et al., 2003; Restivo et al., 2009). Therefore, we analyzed spine density of apical CA1 hippocampal neurons from wild-type and mutant mice. We found that it was significantly higher (14%; *p* < 0.05) in the *Wdr13*^−/0^ mice as compared to the wild-type (Figures 6A–C). We also analyzed dendritic branching of hippocampal CA1 neurons which revealed no significant differences (Figure 6D; ANOVA, *p* > 0.05) between the wild-type and *Wdr13*^−/0^ mice.

To understand the molecular mechanism(s) behind the observed phenotype, iTRAQ based quantitative proteomics was done from hippocampus of *Wdr13*^−/0^ and wild-type mice. Proteomic analysis (Supplementary Sheet 1; Table 2) revealed that 78 proteins were upregulated at greater than 1.5 folds out of a total of 170 quantified (1% FDR) proteins in the *Wdr13*^−/0^ mice. These upregulated proteins included synaptic proteins, namely, SYN1, RAB3A, CAMK2A, and SV2B, proteins belonging to the 14-3-3 family, tubulins, dynamins, etc. In a separate proteomics experiment (data not shown), we found a decrease in protein levels of neurogranin (NRGN) in *Wdr13*^−/0^ hippocampus.

**Table 2:** **Proteins found up-regulated (>1.5 folds) in the hippocampus of ***Wdr13***^**−/0**^ mice as compared to wild-type mice (78 out of 170 quantified)**.

**No**.	**Category**	**Proteins upregulated**
1	Unknown	19 (IPI00831115.3, IPI01027830.1, IPI00986691.1, IPI00753044.1, IPI00775970.1, IPI00974916.1, IPI00473320.2, IPI00649084.1, IPI00990529.1, IPI00110658.1, IPI00831369.1, IPI00475306.1, IPI00987992.1, IPI01026987.1, IPI00648119.1, IPI00762803.1, IPI00265107.4, IPI00830929.1, IPI00970521.1)
2	Synaptic transmission, synaptic vesicle cycling, synapse molecule pathway	16
2A	AMPA cycling	2 (Vesicle-fusing ATPase, Alpha-soluble NSF attachment protein)
2B	Glutamate cycling	3 (Vesicular glutamate transporter 1, Isoform Glt-1B of Excitatory amino acid transporter 2, Calcium-binding mitochondrial carrier protein Aralar1)
2C	GABA metabolism	1 (Isoform 2 of 4-aminobutyrate aminotransferase, mitochondrial)
2D	Cam kinase regulation	4 (Isoform 2 of Neurochondrin, Protein phosphatase 1E, Isoform Alpha CaMKII of Calcium/calmodulin-dependent protein kinase type II subunit alpha, CaM kinase-like vesicle-associated protein)
2E	Synaptic vesicle proton gradient	3 (V-type proton ATPase subunit E 1, V-type proton ATPase subunit d 1, V-type proton ATPase subunit B, brain isoform)
2F	Synaptic	3 (Synaptic vesicle glycoprotein 2B, Dihydropyrimidinase-related protein 2, Isoform Ib of Synapsin-1)
3	Structural	15 (Adenylyl cyclase-associated protein 1, Isoform 2 of Alpha-adducin, Isoform 5 of Dynamin-1, Beta-globin, Tubulin alpha-4A chain, Tubulin alpha-1A chain, Profilin-1, Hemoglobin subunit beta-2, Isoform 3 of Dynamin-1, Tubulin beta-2B chain, Tubulin beta-4 chain, Thy-1 membrane glycoprotein, Isoform 2 of Spectrin alpha chain, brain, 6.8 kDa mitochondrial proteolipid, cofilin-1-like)
4	Protein synthesis	1 (Isoform 3 of Ankyrin repeat and sterile alpha motif domain-containing protein 1B)
5	Metabolism	13 (Phosphoglycerate kinase 1, Cytochrome c oxidase subunit 7A2, mitochondrial, Transaldolase, Thioredoxin-dependent peroxide reductase, mitochondrial, Phosphorylase, Isoform M1 of Pyruvate kinase isozymes M1/M2, Creatine kinase U-type, mitochondrial, Cytochrome b-c1 complex subunit 2, mitochondrial, Isoform 1 of Low molecular weight phosphotyrosine protein phosphatase, Fructose-bisphosphate aldolase A, Isoform 2 of Obg-like ATPase 1, Serine/threonine-protein phosphatase, Cytochrome b-c1 complex subunit 8)
6	Chaperone	2 (Heat shock protein HSP 90-beta, Parkinson disease (Autosomal recessive, early onset) 7)
7	Cell signaling	9 (plasma membrane calcium ATPase 1, NEDD8, Isoform 2 of Serine/threonine-protein phosphatase 2B catalytic subunit alpha isoform, Isoform 2 of 14-3-3 protein theta, 14-3-3 protein zeta/delta, 14-3-3 protein epsilon, 14-3-3 protein gamma, Isoform 2 of Nck-associated protein 1, Gamma-enolase)
8	Cell adhesion and migration	1 (neurocan core protein-like)
9	Vesicular trafficking and fusion	2 (Clathrin heavy chain 1, AP-2 complex subunit alpha-2)

*The classifications have been done manually based on Refseq and Uniprot annotations*.

**Figure 7:**
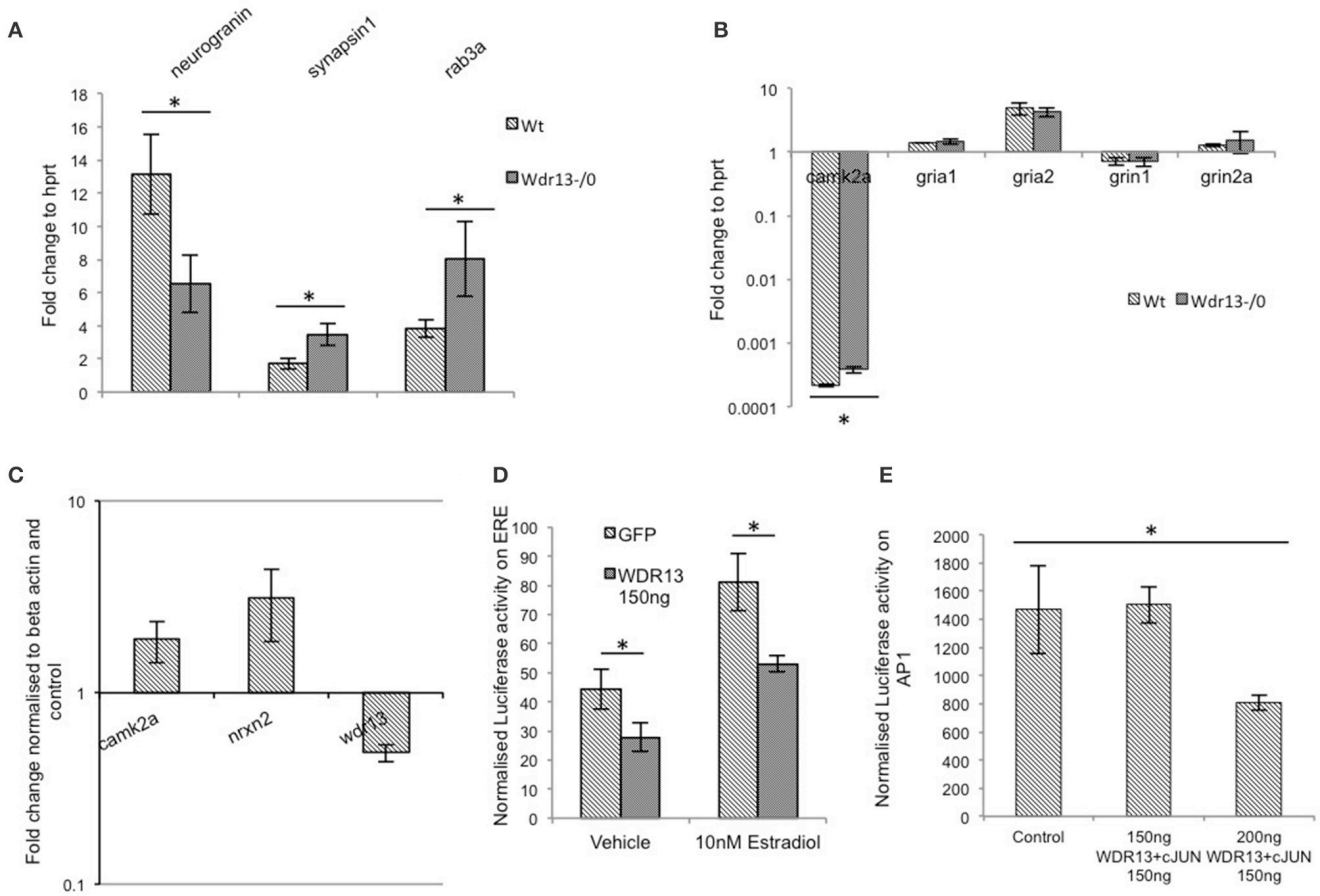
**(A)** In *Wdr13*^−/0^ mice hippocampus, transcript levels of *Syn1* (synapsin1) and *Rab3a* showed increase (*T*-test; *p* < 0.05) and *Nrgn* (neurogranin) showed decrease (*p* < 0.05) than the wild-type under non-stress conditions (*n* = 5) Data represented as ±SEM. **(B)** In *Wdr13*^−/0^ mice, there were no significant changes in levels of *Grin1, Grin2a, Gria1*, and *Gria2* in the hippocampus whereas transcripts of *Camk2a* were upregulated (*n* = 4). Data represented as ±SD. **(C)** Knockdown of *Wdr13* in Neuro2a cells using siRNA resulted in increased transcripts of *Camk2a* and *Nrxn2*. Data represented as ±SD. **(D)** WDR13 repressed luciferase transcription from a promoter containing an ERE element in the presence of Estradiol (Mann Whitney; *p* < 0.05). **(E)** Luciferase activity of AP1 promoter containing vector in Neuro2a cells showed decrease upon co-expression of c-JUN with WDR13 (One way ANOVA; *p* < 0.05). Data represented as ±SD. Wt, wild-type; *Wdr13*^−/0^, *Wdr13* knockout mice. ^*^p < 0.05.

Transcription analysis of *Syn1* (synapsin1) and *Rab3a* (Figure 7A) from hippocampus was consistent with the proteomics data (*p* < 0.05), implying that absence of *Wdr13* gene resulted in upregulation of key synaptic genes in mice. Similarly, *Nrgn* transcription was downregulated (*p* = 0.03). There were, however, no significant changes in the transcript levels of *Grin1, Grin2a* (NMDA receptors) and *Gria1, Gria2* (AMPA receptors) but changes (*p* < 0.05) were observed in transcript levels of *Camk2a* consistent with the proteomics data (Figure 7B). Interestingly, we also found a significant upregulation in transcript levels of immediate early genes *c-Fos* and *Arc*, but not *Bdnf* (Supplementary Figure 5) in hippocampus of *Wdr13*^−/0^ mice when subjected to a novel context (objects placed in an open field) as compared to the wild-type mice.

We wanted to understand whether the changes observed in the expression of multiple synaptic genes were due to the genetic deletion of *Wdr13*. We found that downregulation of *Wdr13* in Neuro2a cells (Supplementary Figures 6A,C) resulted in upregulation of *Camk2a* and *Nrxn2* (Figure 7C), indicating direct effect of absence/levels of WDR13 on the expression of these genes. Previous work carried out in our laboratory has established cJUN, ERα/β, PHIP, and HDACs as interacting partners of WDR13 (V. P. Singh and Shalu Singh, personal communication). We found that in Neuro2a cell line, WDR13 caused repression in transcription (*p* = 0.03) from promoters containing the Estrogen Receptor Element (ERE) (Figure 7D) in both presence and absence of estradiol. Luciferase assay also indicated that co-expression of WDR13 with c-JUN (Supplementary Figures 6A,B,D,E) resulted in repression of transcription from AP1 element containing promoter (Figure 7E; one way ANOVA; *p* < 0.05). We therefore hypothesized that in the absence of *Wdr13*, any repression (competitive or non-competitive) over key genes caused due to its interaction with its partner(s) (Perissi et al., 2010), might be relieved, resulting in their upregulation leading to the observed phenotype.

## Discussion

The current work highlights the action of *Wdr13* in the brain. We showed that WDR13 represses transcription from AP1 and ERE elements containing promoters, which harbor c-JUN and ERα/β responsive elements respectively. Absence of *Wdr13* led to de-regulated expression of multiple genes. Many of these include synaptic genes like *Syn1, Rab3a, Nrxn2, Camk2a*, etc. Interestingly, we showed that absence of *Wdr13* caused mild anxiety and improved retention in MWM task, associated with increased spine density.

We selected the age window of 2–3 months of age to carry out all behavior experiments. There were no systemic changes in the *Wdr13*^−/0^ mice at this age (Singh et al., 2012) and we did not find any changes in brain metabolism. Though we found increased glutamate metabolism at 10 months of age in mutant mice, we argued that this change was mostly neuronal, considering that there were no changes in concentration of 13C glutamate labeled from acetate- indicative of astroglial metabolism. The changes observed at 10 months in *Wdr13*^−/0^ mice might have resulted from accumulating molecular changes in the brain due to the absence of *Wdr13*. Since de-regulation of glutamate can lead to changes in memory (McEntee and Crook, 1993; Gecz, 2010), anxiety (Bergink et al., 2004), depression (Sanacora et al., 2012), etc., it would be interesting to study the behavioral phenotype of *Wdr13*^−/0^ mice at 10 months of age. Increase in glutamate in a chronic state can also lead to excitotoxicity (Foran and Trotti, 2009). Therefore, analysis of brain function and anatomy is important to shed light on the effect of genetic deletion of *Wdr13* in aged mice brain. A conditional knockout mouse with brain specific deletion of *Wdr13* would be more suitable for this age dependent study since systemic metabolic effects because of the absence of this gene could be avoided.

Before analyzing differentially regulated genes, we asked if whether the proliferation of adult neuronal precursor cells was affected using BrDU labeling. Our results showed no significant difference in BrDU positive cells of DG and SVZ between *Wdr13*^−/0^ and wild-type mice unlike pancreatic beta cells. This also indicated that the pathways affected in brain might be different from pancreas or other tissues.

We showed that the absence of *Wdr13* resulted in upregulation of multiple synaptic proteins (16 out of 78 upregulated) *in-vivo*. We showed similar results in downregulation of *Wdr13* in Neuro2a cell lines indicating that the changes occurred as a consequence of loss of the gene and not due to any systemic effects. The synaptic proteins found upregulated could be classified into AMPA cycling, Glutamate cycling, GABA metabolism, CAM kinase regulation, Synaptic vesicle proton gradient and synaptic vesicle cycling (Table 1). We observed notable upregulation in *Camk2a, Syn1*, and *Rab3a* levels in *Wdr13*^−/0^ mice at transcript and protein levels. It is known that synapsins control the release of neurotransmitters such as glutamate and important for synaptic plasticity (Nichols et al., 1992; Jovanovic et al., 2000). Synapsins are involved in memory formation and consolidation process in drosophila and in aging-related memory impairment in mammals (Godenschwege et al., 2004; John et al., 2009). Similarly, RAB3A is important for synaptic transmission, learning and memory (Yang et al., 2007). CAMK2A belongs to the CaM-kinase II family of proteins-known for their significant role in synaptic plasticity, long term potentiation (LTP), and learning and memory (Soderling, 1993). Levels of Vesicular glutamate transporter 1 (VGLUT1)—an important molecule for excitatory synapse and for maintaining LTP (Balschun et al., 2010) was also found to be increased in *Wdr13*^−/0^ mice. Upregulation was also recorded in the levels of 14-3-3 proteins in *Wdr13*^−/0^ mice. 14-3-3 proteins are positive regulators of associative learning and memory (Qiao et al., 2014). An upregulation in dynamins and tubulins found in the *Wdr13*^−/0^ mice could be important factors behind the increased spine density observed (Gray et al., 2005; Shirao and González-Billault, 2013). Since spine density has been related to learning and memory, upregulation of afore-mentioned proteins might be of significance regarding the observed phenotype. We also found upregulation of immediate early genes *Arc* and *c-Fos* in *Wdr13*^−/0^ mice when exposed to a novel environment as compared to wild-type mice. Arc has traditionally been associated with learning and memory, particularly long term spatial memory (Bramham et al., 2010). C-Fos has also been shown to be upregulated during learning trials (Alberini, 2009). It may be noted that no changes were found in the transcript levels of NMDA and AMPA receptor genes. However, considering that the levels of multiple synaptic proteins including important proteins like CAMK2A and immediate early genes like *Arc*, were found to be upregulated, an increased LTP might be expected to be associated with the phenotype of better spatial memory in *Wdr13*^−/0^ mice. These findings may be corroborated with the help of further experiments particularly electrophysiology. Thus, taken together, increase in levels of above mentioned synaptic proteins and increased synaptic activity as measured by *Arc* and *c-Fos*, might be few of the key factors responsible for better performance in spatial memory task of MWM by *Wdr13*^−/0^ mice.

Interestingly, many of the synaptic proteins along with Clathrin, AP-2, and Dynamin found up-regulated in *Wdr13*^−/0^ mice are also known to be important for synaptic vesicle recycling. Synaptic vesicle recycling is essential for synaptic plasticity, memory, and cognitive ability, and any deleterious changes in it lead to mental retardation, schizophrenia, and defects in spatial memory (Murthy and De Camilli, 2003; Glyvuk et al., 2010; Cottrell et al., 2013). Synaptic recycling affects synaptic transmission (Casillas-Espinosa et al., 2012) and consequently memory through LTP (Hölscher, 1999). In this context it would be interesting to investigate synaptic recycling in these mutant mice.

Though *Wdr13*^−/0^ mice showed better spatial memory, they didn't show any differences in spatial learning as assessed using MWM than that of the wild-type mice. Previous reports show that disruption of a gene can affect memory without significantly changing learning ability (Maguschak and Ressler, 2008; Tsai et al., 2012) though the molecular reasons are not clearly understood.

*Wdr13*^−/0^ mice also showed decreased exploration of central area of OFT and active avoidance of novel object but failed to show any significant difference in EPM, particularly in CD1 background. It is possible that the response of the mutant mice was directed specifically against novel environment (Bailey and Crawley, 2009) and therefore differences were observed in OFT and NOR. Since the mutant mice also traversed significantly more distance, it explored EPM as much as the wild-type mice leading to no observable differences in CD1 background. However, it is to be noted that mutant mice in C57Bl/6J background showed a trend of increased visit to closed arm of EPM, and therefore, it is possible that this phenotype of mild novelty-associated anxiety was affected by the genetic background (CD1).

In our analysis, we found decreased levels of Neurogranin (*Nrgn*) at protein and transcript levels in *Wdr13*^−/0^ mice. Neurogranin is a brain-specific calmodulin-binding protein expressed particularly in dendritic spines. *Nrgn*^−/−^ mice exhibit characteristics of anxiety (Miyakawa et al., 2001). Similarly, it has been shown that overexpression of *Camk2a* leads to increased anxiety in mice (Hasegawa et al., 2009).

It is known through previous studies carried out in our lab that WDR13 interacts with c-JUN, Estrogen receptors ERα/β and HDACs (V. P. Singh and Shalu Singh, personal communication). Consistent with our previous findings, we show that WDR13 represses transcription from promoter containing ERE (ERα/β responsive) and AP1 (c-JUN responsive) elements. The action leading to the observed phenotype and deregulation of the multiple afore-mentioned genes in *Wdr13*^−/0^ mice might have been attained by relieving of competitive repression (Perissi et al., 2010) induced by WDR13 on downstream targets of c-JUN and ERα/β—both shown to be positive regulators of learning and memory genes. Learning trials are known to increase *cJun* levels which affect learning positively by altering the expression of downstream genes (Alberini, 2009). While estrogen aids synaptic transmission and plasticity by positively regulating synaptic genes (Foy et al., 2010), activation of Estrogen receptors like ERβ has also been shown to improve memory (Liu et al., 2008). Further, estrogen induces synaptic protein CAMK2A activity *in-vivo* (Sambrook and Russell, 2001). Additionally, administration of estradiol increases transcription of *Syn1* (Pan et al., 2010). *Syn1* has also been reported to contain an ERE element upstream (Bourdeau et al., 2004) indicating that the action of estrogen on its transcription is guided through Estrogen receptors. Interestingly, in our analysis we have found both *Camk2a* and *Syn1* to be upregulated in *Wdr13*^−/0^ mice.

Multiple genes that affect memory and learning have been reported in previous studies. Modulation of expression of a number of them had resulted in better learning and memory. These genes affect different processes like excitatory synaptic transmission (*Nr2b, Cdk5, p25, Hgf* etc.), inhibitory synaptic transmission (GABA receptor, *Grpr, Pkr* etc.), pre-synaptic function (*H-ras, Ncx2, Cbl-b*, etc.), transcriptional regulation (*CREB, CamkIV, Gcn2, Calcineurin*, etc.), miRNA biogenesis (*Dicer1*), epigenetic regulation (*Hdac2*) etc. (Lee, 2014). Our results show that absence of *Wdr13* in 2 months old mice leads to better spatial memory associated with upregulation of multiple synaptic proteins. While it is exciting to find a molecule, manipulation of which may enhance memory, long-term implications of removal of this gene needs to be studied. As mentioned earlier increased glutamate can lead to excitotoxicity and neuronal death and hence aged mice lacking *Wdr13* should be analyzed. Also, the persistent increase in synaptic proteins has been associated with schizophrenia (Li et al., 2015). Hence, it should be investigated whether absence of *Wdr13* leads to hallucinogenic or schizophrenia like effect in mice with increasing age.

## Author contributions

SM designed and executed the experiments and drafted the manuscript. GS executed with SM proteomics experiments. VT with SM executed NMR experiments. BL helped in animal breeding, maintenance, and genotyping. ST supervised the proteomics experiment providing inputs and helping in design, execute and in acquisition of the results for the experiment. SK gave inputs, aided in interpreting the data, revised the draft manuscript and finalized it.

### Conflict of interest statement

The authors declare that the research was conducted in the absence of any commercial or financial relationships that could be construed as a potential conflict of interest. The reviewer SB and handling Editor declared their shared affiliation, and the handling Editor states that the process nevertheless met the standards of a fair and objective review.

## Supplementary material

Supplementary Figure 1 **RNA ***in situ*** hybridization of ***Wdr13*** in the brain. (A)** RISH using antisense *Wdr13* probe showed localization of it's transcript in Hippocampal CA1, CA2, CA3 region, and Dentate Gyrus, and in Cortex. The **left** panel depicts antisense probe and **right** panel depicts sense probe. Scale 1 mm. **(B)** Antisense *Wdr13* probing (top) of a sagittal section of mouse brain showing significant hybridization in Cerebellum, Hippocampus, and Cortex. Scale 1 mm. **(C)** A coronal section of cortex hybridized with anti-sense *Wdr13* probe. Scale 200 μm.

Supplementary Figure 2 **Histological staining of brain sections from ***Wdr13***^**−/0**^ and wild-type mice showed no significant differences (***n*** = 3). (A)** Nissl staining of a coronal section of brain showing the hippocampus. Wild-type is shown on left and *Wdr13*^−/0^ on right. **(B)** Nissl staining of sagittal section of brain. Wild-type is shown in top and *Wdr13*^−/0^ in bottom panel. **(C)** H&E staining of a coronal section of brain. Wild-type on left and *Wdr13*^−/0^ on right. **(D)** H&E staining of sagittal section of brain. Wild-type on top and *Wdr13*^−/0^ bottom panel. Scale 1 mm.

Supplementary Figure 3 **Additional dataset: Behavioral analysis of ***Wdr13***^**−/0**^ and wild-type mice in CD1 background (Cohort 1). (A)** Open field test. *Wdr13*^−/0^ mice showed marginally (*T*-test; *p* = 0.07) decreased exploration time in the central area of the open field as compared to the wild-types. **(B)** Novel object recognition test. *Wdr13*^−/0^ mice showed a trend (*T*-test; *p* = 0.06) of decreased exploration of novel object than the familiar object (*n* = 5). **(C)** Elevated plus maze test. There were no significant differences (*T*-test; *p* > 0.05) between *Wdr13*^−/0^ and wild-type mice in total time spent in closed or open arms (*n* = 5). **(D)** Morris water maze test. *Wdr13*^−/0^ mice spent significantly more time [ANOVA, *F*_(1, 56)_ = 5.44; *p* < 0.05] in the target quadrant during extinction trials as compared to the wild-type mice (*n* = 5). Data represented as ±SEM. Wt, wild-type; *Wdr13*^−/0^, *Wdr13* knockout mice. ^*^*p* < 0.05.

Supplementary Figure 4 **Tracking adult neuronal proliferation in ***Wdr13***^**−/0**^ and wild-type mice using BrDU labeling. (A)** Representative images of fluorescent labeling for anti-BrDU in wild-type and *Wdr13*^−/0^ Dentate Gyrus (DG). **(A**′**)** There was no significant difference (*T*-test; *p* > 0.05) in the number of BrDU labeled cells from the DG of hippocampus. **(B)** Representative images of Dab staining for anti-BrDU labeling in wild-type and *Wdr13*^−/0^ Sub Ventricular Zone (SVZ). **(B**′**)** There was no significant difference (*T*-test; *p* > 0.05) in the number of BrDU labeled cells from SVZ of *Wdr13*^−/0^ and wild-type mice. **(C)** There were no significant differences in number of BrDU positive cells in hippocampal DG and in **(D)**. SVZ of *Wdr13*^−/0^ mice as compared to the wild-type mice after performing learning task in Morris water maze. Data represented as ±SEM. Wt, wild-type; *Wdr13*^−/0^, *Wdr13* knockout mice.

Supplementary Figure 5 **Real time analysis of hippocampus from ***Wdr13***^**−/0**^ and wild-type mice which were exposed to a novel environment revealed increased transcripts of *c-Fos* and *Arc* in *Wdr13*^−/0^ hippocampus**. However, there was no significant difference (*T*-test; *p* > 0.05) in transcript levels of *Bdnf* (*n* = 4). Data is represented ±SD. Wt, wild-type; *Wdr13*^−/0^, *Wdr13* knockout mice.

Supplementary Figure 6 **Western analysis. (A)** Representative blot of WDR13 overexpression and knockdown (from left) in Neuro2a cells. **(B)** Relative quantification of WDR13 overexpression (Mann Whitney; *p* < 0.05) and knockdown (Mann Whitney; *p* < 0.05). **(C)** Percent relative expression (compared to controls) for WDR13 knockdown and overexpression. **(D)** Representative blot and **(E)** relative quantification (triplicates) of c-JUN overexpression (Mann Whitney; *p* < 0.05) in Neuro2a cell line. Data is represented ±SD.
