# Supplementary figures and images for: Implication of Genetic Deletion of Wdr13 in Mice: Mild Anxiety, Better Performance in Spatial Memory Task, with Upregulation of Multiple Synaptic Proteins
Source: Front Mol Neurosci. 2016 Aug 30;9:73. doi: 10.3389/fnmol.2016.00073 (PMC5003927; doi:10.3389/fnmol.2016.00073)

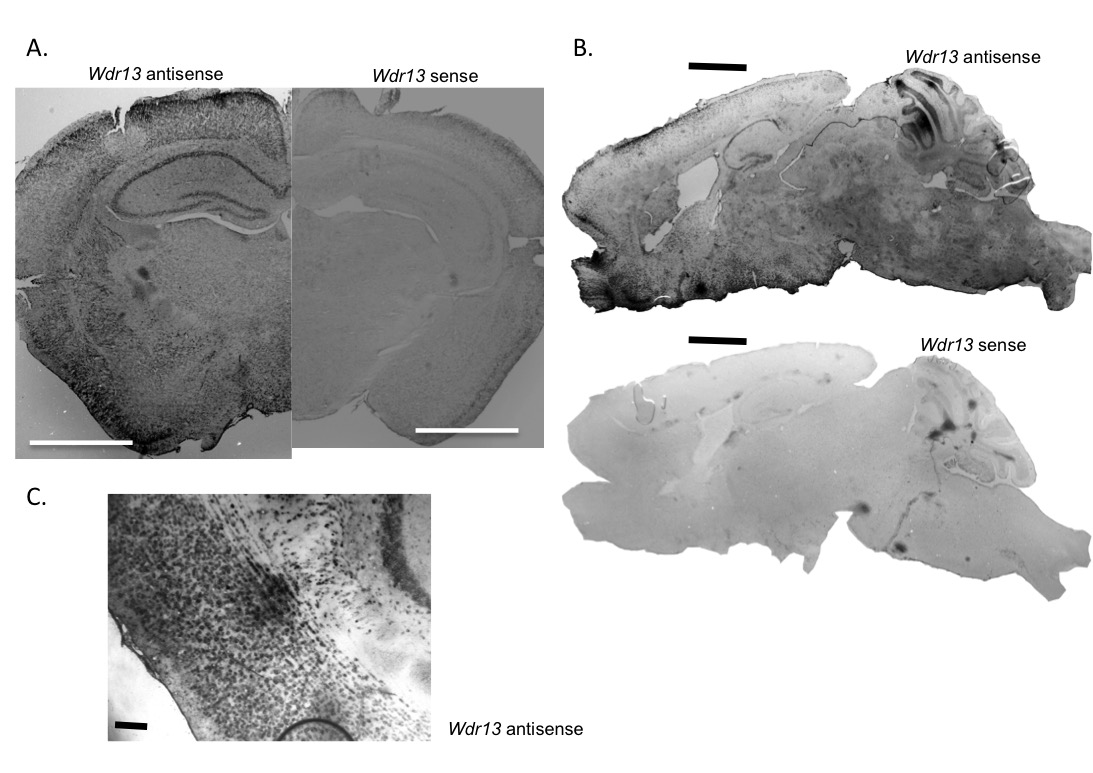

Supplement: Supplementary Figure 1 — RNA in situ hybridization of Wdr13 in the brain. (A) RISH using antisense Wdr13 probe showed localization of it's transcript in Hippocampal CA1, CA2, CA3 region, and Dentate Gyrus, and in Cortex. The left panel depicts antisense probe and right panel depicts sense probe. Scale 1 mm. (B) Antisense Wdr13 probing (top) of a sagittal section of mouse brain showing significant hybridization in Cerebellum, Hippocampus, and Cortex. Scale 1 mm. (C) A coronal section of cortex hybridized with anti-sense Wdr13 probe. Scale 200 μm. [file Image1.JPEG]

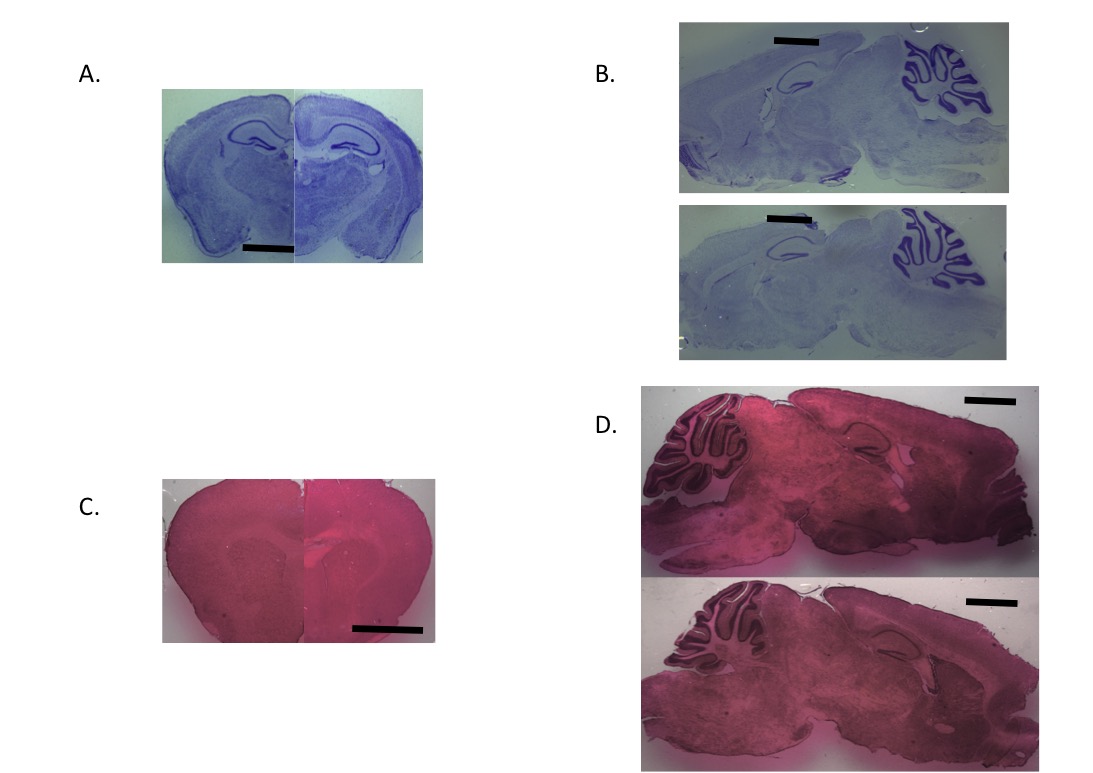

Supplement: Supplementary Figure 2 — Histological staining of brain sections from Wdr13−/0 and wild-type mice showed no significant differences (n = 3). (A) Nissl staining of a coronal section of brain showing the hippocampus. Wild-type is shown on left and Wdr13−/0 on right. (B) Nissl staining of sagittal section of brain. Wild-type is shown in top and Wdr13−/0 in bottom panel. (C) H&E staining of a coronal section of brain. Wild-type on left and Wdr13−/0 on right. (D) H&E staining of sagittal section of brain. Wild-type on top and Wdr13−/0 bottom panel. Scale 1 mm. [file Image2.JPEG]

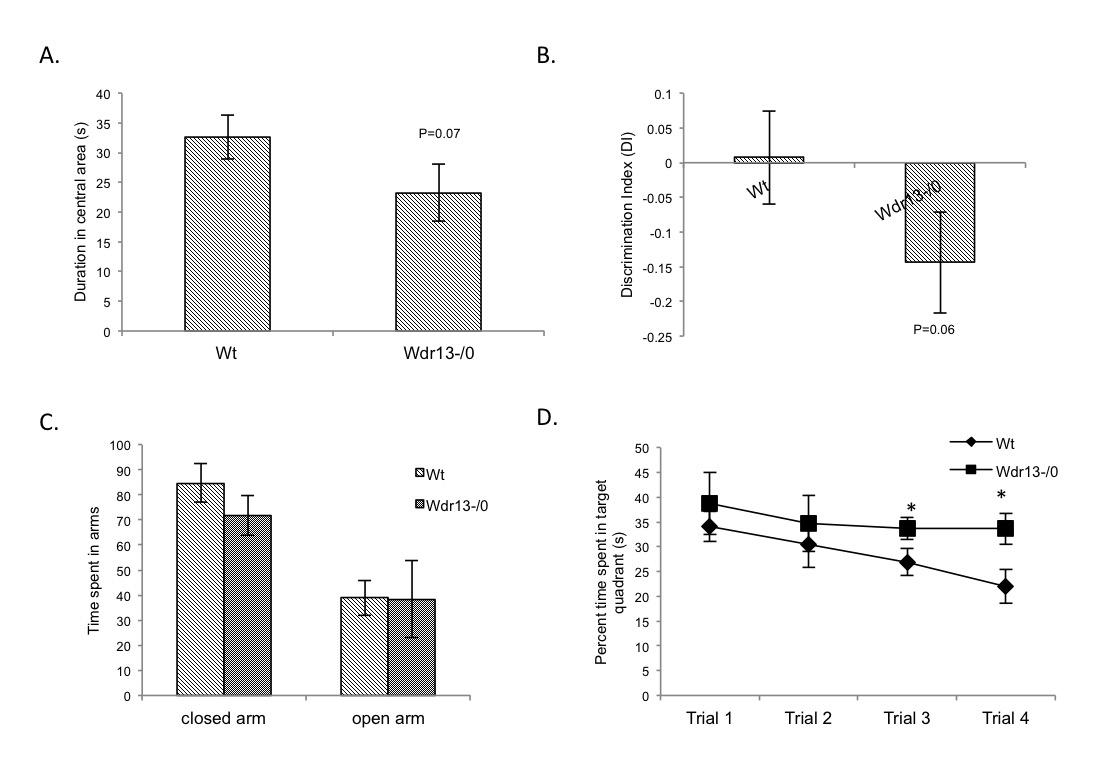

Supplement: Supplementary Figure 3 — Additional dataset: Behavioral analysis of Wdr13−/0 and wild-type mice in CD1 background (Cohort 1). (A) Open field test. Wdr13−/0 mice showed marginally (T-test; p = 0.07) decreased exploration time in the central area of the open field as compared to the wild-types. (B) Novel object recognition test. Wdr13−/0 mice showed a trend (T-test; p = 0.06) of decreased exploration of novel object than the familiar object (n = 5). (C) Elevated plus maze test. There were no significant differences (T-test; p > 0.05) between Wdr13−/0 and wild-type mice in total time spent in closed or open arms (n = 5). (D) Morris water maze test. Wdr13−/0 mice spent significantly more time [ANOVA, F(1, 56) = 5.44; p < 0.05] in the target quadrant during extinction trials as compared to the wild-type mice (n = 5). Data represented as ±SEM. Wt, wild-type; Wdr13−/0, Wdr13 knockout mice. *p < 0.05. [file Image3.JPEG]

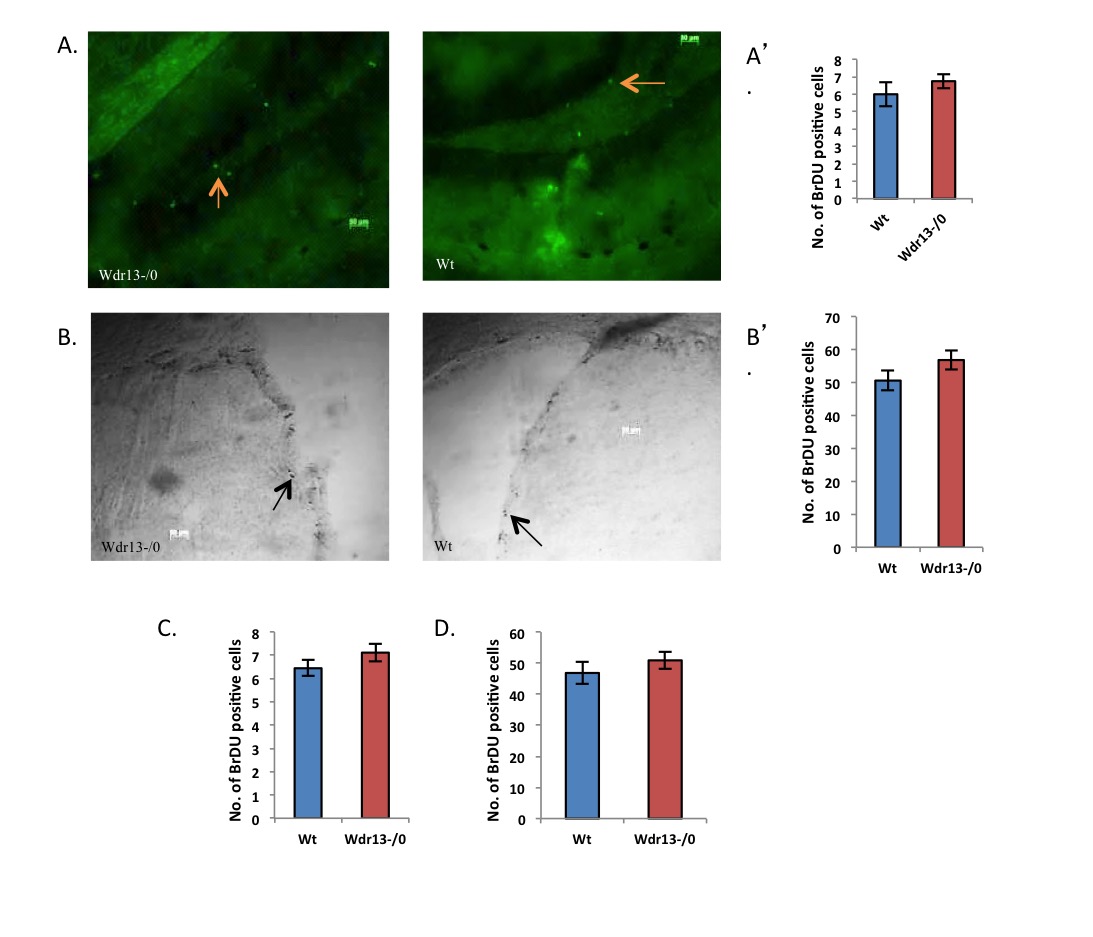

Supplement: Supplementary Figure 4 — Tracking adult neuronal proliferation in Wdr13−/0 and wild-type mice using BrDU labeling. (A) Representative images of fluorescent labeling for anti-BrDU in wild-type and Wdr13−/0 Dentate Gyrus (DG). (A′) There was no significant difference (T-test; p > 0.05) in the number of BrDU labeled cells from the DG of hippocampus. (B) Representative images of Dab staining for anti-BrDU labeling in wild-type and Wdr13−/0 Sub Ventricular Zone (SVZ). (B′) There was no significant difference (T-test; p > 0.05) in the number of BrDU labeled cells from SVZ of Wdr13−/0 and wild-type mice. (C) There were no significant differences in number of BrDU positive cells in hippocampal DG and in (D). SVZ of Wdr13−/0 mice as compared to the wild-type mice after performing learning task in Morris water maze. Data represented as ±SEM. Wt, wild-type; Wdr13−/0, Wdr13 knockout mice. [file Image4.JPEG]

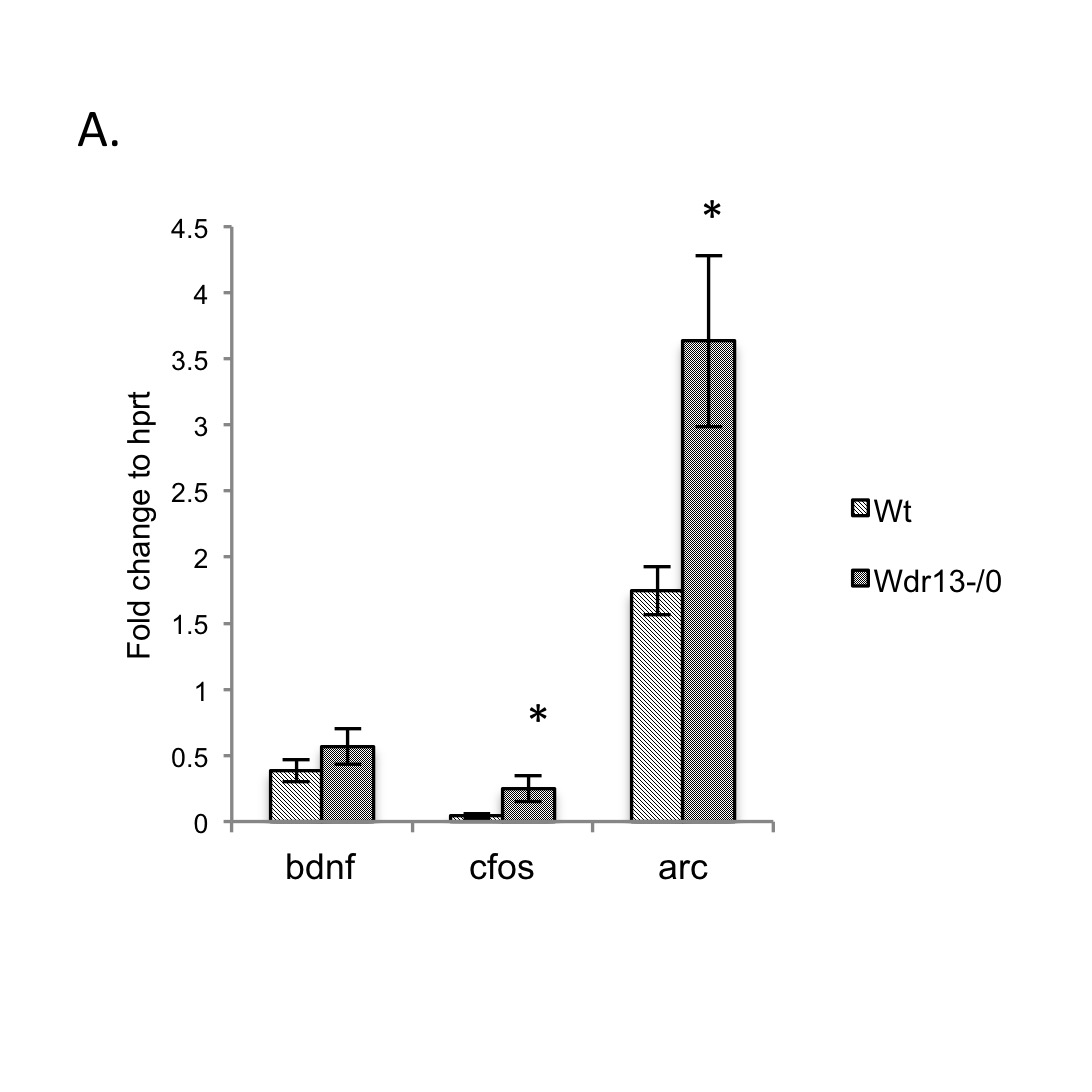

Supplement: Supplementary Figure 5 — Real time analysis of hippocampus from Wdr13−/0 and wild-type mice which were exposed to a novel environment revealed increased transcripts of c-Fos and Arc in Wdr13−/0 hippocampus. However, there was no significant difference (T-test; p > 0.05) in transcript levels of Bdnf (n = 4). Data is represented ±SD. Wt, wild-type; Wdr13−/0, Wdr13 knockout mice. [file Image5.JPEG]

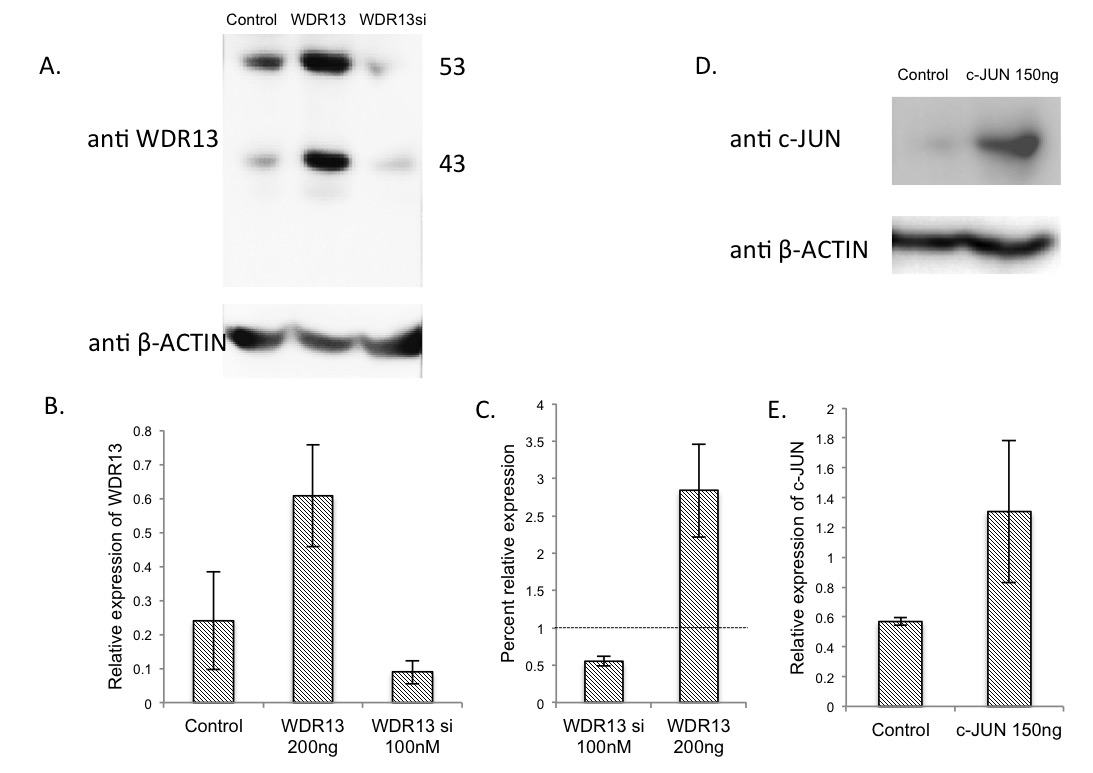

Supplement: Supplementary Figure 6 — Western analysis. (A) Representative blot of WDR13 overexpression and knockdown (from left) in Neuro2a cells. (B) Relative quantification of WDR13 overexpression (Mann Whitney; p < 0.05) and knockdown (Mann Whitney; p < 0.05). (C) Percent relative expression (compared to controls) for WDR13 knockdown and overexpression. (D) Representative blot and (E) relative quantification (triplicates) of c-JUN overexpression (Mann Whitney; p < 0.05) in Neuro2a cell line. Data is represented ±SD. [file Image6.JPEG]
