# Supplementary material for: Implication of Genetic Deletion of Wdr13 in Mice: Mild Anxiety, Better Performance in Spatial Memory Task, with Upregulation of Multiple Synaptic Proteins
Source: Front Mol Neurosci. 2016 Aug 30;9:73. doi: 10.3389/fnmol.2016.00073 (PMC5003927; doi:10.3389/fnmol.2016.00073)
Supplement: Supplementary file 7 [file Table1.PDF]

SUPPLEMENTARY SHEET 1		
Accession	Description	Wdr13-/0(KO)/Wt
IPI00831115.3	Uncharacterized protein	**2.518**
IPI00137331.7	Adenylyl cyclase-associated protein 1	**2.308**
IPI00656325.2	Vesicle-fusing ATPase	**2.203**
IPI00225322.1	Isoform 2 of Alpha-adducin	**2.195**
IPI00753037.5	Isoform 5 of Dynamin-1	**2.114**
IPI00555069.3	Phosphoglycerate kinase 1	**2.060**
IPI00109153.7	Vesicular glutamate transporter 1	**2.010**
IPI01027830.1	9 kDa protein	1.997
IPI00762198.2	Beta-globin	1.976
IPI00117350.1	Tubulin alpha-4A chain	1.970
IPI00114377.1	Cytochrome c oxidase subunit 7A2, mitochondrial	1.959
IPI00889265.1	Isoform 2 of Neurochondrin	1.938
IPI00119115.2	V-type proton ATPase subunit E 1	1.923
IPI00986691.1	Uncharacterized protein	1.919
IPI00554929.3	Heat shock protein HSP 90-beta	1.904
IPI00110753.1	Tubulin alpha-1A chain	1.903
IPI00224740.6	Profilin-1	1.894
IPI00124692.1	Transaldolase	1.870
IPI00461286.1	Protein phosphatase 1E	1.854
IPI00316491.4	Hemoglobin subunit beta-2	1.844
IPI00753044.1	20 kDa protein	1.834
IPI00465648.3	Isoform 3 of Dynamin-1	1.813
IPI00775970.1	Uncharacterized protein	1.784
IPI00556827.1	plasma membrane calcium ATPase 1	1.776
IPI00116192.1	Thioredoxin-dependent peroxide reductase, mitochondrial	1.772
IPI00127021.1	NEDD8	1.759
IPI00856665.1	Phosphorylase	1.752
IPI00673967.2	Isoform 3 of Ankyrin repeat and sterile alpha motif domain-contain	1.751
IPI00109061.1	Tubulin beta-2B chain	1.749
IPI00974916.1	Uncharacterized protein (Fragment)	1.726
IPI00473320.2	Putative uncharacterized protein	1.722
IPI00109073.5	Tubulin beta-4 chain	1.722
IPI00621806.2	Isoform Alpha CaMKII of Calcium/calmodulin-dependent protein kir	1.704
IPI00756703.1	Isoform 2 of Serine/threonine-protein phosphatase 2B catalytic sub	1.692
IPI00845840.1	Isoform M1 of Pyruvate kinase isozymes M1/M2	1.675
IPI00649084.1	Uncharacterized protein	1.672
IPI00230290.1	Isoform Glt-1B of Excitatory amino acid transporter 2	1.672
IPI00128296.1	Creatine kinase U-type, mitochondrial	1.654
IPI00990529.1	Uncharacterized protein (Fragment)	1.653
IPI00313841.1	V-type proton ATPase subunit d 1	1.652
IPI00110658.1	Putative uncharacterized protein	1.640
IPI00118930.1	Alpha-soluble NSF attachment protein	1.639
IPI00221456.1	Synaptic vesicle glycoprotein 2B	1.625
IPI00119138.1	Cytochrome b-c1 complex subunit 2, mitochondrial	1.602
IPI00990803.1	neurocan core protein-like	1.596

IPI00134135.1	Isoform 1 of Low molecular weight phosphotyrosine protein phosph	1.592
IPI00656269.1	Isoform 2 of 14-3-3 protein theta	1.591
IPI00169916.11	Clathrin heavy chain 1	1.590
IPI00831369.1	MKIAA4049 protein	1.585
IPI00116498.1	14-3-3 protein zeta/delta	1.582
IPI00221402.7	Fructose-bisphosphate aldolase A	1.580
IPI00109727.1	Thy-1 membrane glycoprotein	1.575
IPI00475306.1	Putative uncharacterized protein	1.571
IPI00987992.1	Uncharacterized protein	1.567
IPI00114375.2	Dihydropyrimidinase-related protein 2	1.562
IPI01026987.1	16 kDa protein	1.554
IPI00227836.1	Isoform 2 of Obg-like ATPase 1	1.552
IPI00473972.1	Serine/threonine-protein phosphatase	1.552
IPI00118384.1	14-3-3 protein epsilon	1.550
IPI00753793.2	Isoform 2 of Spectrin alpha chain, brain	1.549
IPI00656204.1	Isoform 2 of Nck-associated protein 1	1.549
IPI00119113.3	V-type proton ATPase subunit B, brain isoform	1.548
IPI00308162.3	Calcium-binding mitochondrial carrier protein Aralar1	1.546
IPI00136372.3	Isoform Ib of Synapsin-1	1.543
IPI00227445.1	Isoform 2 of 4-aminobutyrate aminotransferase, mitochondrial	1.541
IPI00895414.1	Parkinson disease (Autosomal recessive, early onset) 7	1.538
IPI00648119.1	Uncharacterized protein	1.536
IPI00310131.6	AP-2 complex subunit alpha-2	1.531
IPI00762803.1	Uncharacterized protein	1.528
IPI00265107.4	Uncharacterized protein	1.527
IPI00113143.1	6.8 kDa mitochondrial proteolipid	1.527
IPI00224210.5	Cytochrome b-c1 complex subunit 8	1.517
IPI00830929.1	Uncharacterized protein	1.514
IPI00122486.3	CaM kinase-like vesicle-associated protein	1.502
IPI00331704.7	Gamma-enolase	1.502
IPI00848816.1	cofilin-1-like	1.501
IPI00970521.1	Uncharacterized protein	1.500
IPI00230707.6	14-3-3 protein gamma	1.500
IPI00112139.1	OCIA domain-containing protein 2	1.488
IPI00230192.5	Isoform Alpha-1 of Guanine nucleotide-binding protein G(o) subuni	1.486
IPI00830313.1	Uncharacterized protein	1.486
IPI00555118.4	Isoform 2 of Sodium-driven chloride bicarbonate exchanger	1.483
IPI00341282.2	ATP synthase subunit b, mitochondrial	1.481
IPI00750142.2	Uncharacterized protein	1.481
IPI00457898.3	Phosphoglycerate mutase 1	1.478
IPI00928559.1	Putative uncharacterized protein	1.471
IPI00880812.1	Uncharacterized protein	1.464
IPI00117352.1	Tubulin beta-5 chain	1.460
IPI00776166.1	Uncharacterized protein	1.456
IPI00121443.1	Cytochrome c oxidase subunit 6A, mitochondrial	1.454
IPI00230610.2	Isoform DM-20 of Myelin proteolipid protein	1.442
IPI00279443.1	Heat shock 70 kDa protein 12A	1.441

IPI00649695.1	Uncharacterized protein	1.440
IPI00620582.2	Isoform 2 of Actin-related protein 3B	1.439
IPI00468481.2	ATP synthase subunit beta, mitochondrial	1.439
IPI00830211.1	Uncharacterized protein	1.435
IPI00874456.1	Dihydrolipoyl dehydrogenase, mitochondrial	1.433
IPI00857439.1	ATP synthase subunit alpha	1.432
IPI00985596.1	hemoglobin subunit beta-1-like isoform 5	1.431
IPI00228633.7	Glucose-6-phosphate isomerase	1.428
IPI00136703.1	Creatine kinase B-type	1.426
IPI00131896.1	Brain protein 44	1.418
IPI00896727.1	Cullin-associated NEDD8-dissociated protein 1	1.417
IPI00845772.1	Isoform 2 of Cytochrome c1, heme protein, mitochondrial	1.413
IPI00622911.1	Isoform B of AP-2 complex subunit alpha-1	1.406
IPI00756061.1	49 kDa protein	1.403
IPI00988275.1	Glyceraldehyde-3-phosphate dehydrogenase (Fragment)	1.402
IPI00323592.2	Malate dehydrogenase, mitochondrial	1.399
IPI00407339.7	Histone H4	1.393
IPI00128346.1	CDGSH iron-sulfur domain-containing protein 1	1.385
IPI00831553.1	Uncharacterized protein	1.377
IPI00230351.1	Succinate dehydrogenase [ubiquinone] flavoprotein subunit, mitoch	1.375
IPI00114279.1	Excitatory amino acid transporter 1	1.373
IPI00229598.4	Isoform CNPI of 2',3'-cyclic-nucleotide 3'-phosphodiesterase	1.372
IPI00990815.1	alpha-enolase-like isoform 12	1.372
IPI00112251.1	Tubulin beta-3 chain	1.371
IPI00116843.1	NADH-ubiquinone oxidoreductase chain 4	1.369
IPI00117312.1	Aspartate aminotransferase, mitochondrial	1.368
IPI00223596.1	Isoform 13 of Myelin basic protein	1.368
IPI00113141.1	Citrate synthase, mitochondrial	1.365
IPI00988665.1	glyceraldehyde-3-phosphate dehydrogenase-like	1.363
IPI00990063.1	Uncharacterized protein	1.362
IPI00230540.1	Isoform Mt-VDAC1 of Voltage-dependent anion-selective channel p	1.360
IPI00988842.1	Uncharacterized protein	1.359
IPI00886245.1	Uncharacterized protein	1.357
IPI00261627.1	Succinyl-CoA ligase [ADP-forming] subunit beta, mitochondrial	1.350
IPI00608078.1	Isoform 2 of Isocitrate dehydrogenase [NAD] subunit alpha, mitoch	1.343
IPI00313962.3	Ubiquitin carboxyl-terminal hydrolase isozyme L1	1.340
IPI01008458.1	Putative uncharacterized protein	1.338
IPI00114642.4	Histone H2B type 1-F/J/L	1.336
IPI00653231.1	sideroflexin-3 isoform 3	1.331
IPI00850934.2	Uncharacterized protein (Fragment)	1.327
IPI00221528.1	Beta-actin-like protein 2	1.325
IPI00131695.3	Serum albumin	1.323
IPI00420426.1	AP-3 complex subunit beta-2	1.319
IPI00120212.2	NADH dehydrogenase [ubiquinone] 1 alpha subcomplex subunit 9,	1.319
IPI00130920.2	Microtubule-associated protein 1B	1.318
IPI00465810.3	Synaptic vesicle glycoprotein 2A	1.305
IPI00115564.5	ADP/ATP translocase 1	1.303

IPI00886201.1	Uncharacterized protein	1.303
IPI00974762.1	Uncharacterized protein	1.288
IPI00132390.5	NADH dehydrogenase [ubiquinone] 1 beta subcomplex subunit 4	1.285
IPI00754398.1	L-lactate dehydrogenase	1.275
IPI00170093.3	NADH dehydrogenase [ubiquinone] iron-sulfur protein 8, mitochon	1.273
IPI00229527.8	Leukotriene A-4 hydrolase	1.273
IPI00124771.1	Phosphate carrier protein, mitochondrial	1.268
IPI00886297.1	Uncharacterized protein	1.257
IPI00987580.1	Peptidyl-prolyl cis-trans isomerase	1.250
IPI01026712.1	30 kDa protein	1.247
IPI00122048.2	Sodium/potassium-transporting ATPase subunit alpha-3	1.239
IPI00122928.1	Tubulin beta-6 chain	1.238
IPI00900395.2	Uncharacterized protein (Fragment)	1.234
IPI00116753.4	Electron transfer flavoprotein subunit alpha, mitochondrial	1.224
IPI00918086.1	Uncharacterized protein	1.221
IPI00420569.2	Sodium/potassium-transporting ATPase subunit alpha-2	1.216
IPI00661241.1	Isoform 2 of 6-phosphofructokinase type C	1.216
IPI00662028.1	Isoform 2 of Electrogenic sodium bicarbonate cotransporter 1	1.156
IPI00229510.5	L-lactate dehydrogenase B chain	1.154
IPI00131176.1	Cytochrome c oxidase subunit 2	1.135
IPI00785410.1	Cytochrome c oxidase subunit 5B, mitochondrial	1.124
IPI00742400.1	Isoform 2 of cAMP-dependent protein kinase catalytic subunit beta	1.093
IPI00121534.11	Carbonic anhydrase 2	1.091
IPI00881473.1	Uncharacterized protein	1.085
IPI00649645.1	Uncharacterized protein	1.064
IPI00759940.3	Isoform Cytoplasmic of Fumarate hydratase, mitochondrial	1.063
IPI00283761.1	Uncharacterized protein	1.040
IPI00222125.1	Catechol O-methyltransferase domain-containing protein 1	1.038
IPI00985961.1	Uncharacterized protein	1.005
IPI00229544.3	Histone H2A type 1-H	0.970
IPI00553538.3	Histone H3.1	0.861
IPI00114593.1	Actin, alpha cardiac muscle 1	
IPI00120295.1	Claudin-11	
IPI00756073.1	Uncharacterized protein	
IPI00969847.1	Uncharacterized protein	
IPI00856556.1	Uncharacterized protein	
